# Understanding the role of conjugation length on the self-assembly behaviour of oligophenyleneethynylenes†

**DOI:** 10.1039/d1cc01054a

**Published:** 2021-04-27

**Authors:** Beatriz Matarranz, Goutam Ghosh, Ramesh Kandanelli, Angel Sampedro, Kalathil K. Kartha, Gustavo Fernández

**Affiliations:** Organisch-Chemisches Institut, Universität Münster, Corrensstraße 36 Münster 48149 Germany kartha@uni-muenster.de fernandg@uni-muenster.de; Institut für Organische Chemie, Universität Würzburg am Hubland Würzburg 97074 Germany

## Abstract

Oligophenyleneethynylenes (OPEs) are prominent building blocks with exciting optical and supramolecular properties. However, their generally small spectroscopic changes upon aggregation make the analysis of their self-assembly challenging, especially in the absence of additional hydrogen bonds. Herein, by investigating a series of OPEs of increasing size, we have unravelled the role of the conjugation length on the self-assembly properties of OPEs.

## Abstract 2

We unravel the relationship between conjugation length and self-assembly behaviour of oligophenyleneethynylenes (OPEs).

## Graphical abstract

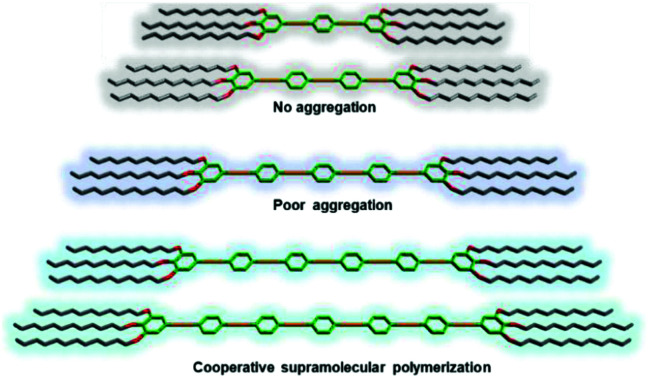

Self-assembled structures of π-conjugated systems have become an important class of functional materials with manifold potential applications ranging from optoelectronics to life sciences.^1^ Particularly, linear oligomeric π-systems have attracted widespread attention by virtue of their exceptional optical and electronic properties.^2^ Examples thereof include oligophenylenes (OPs),^3^ oligophenylenevinylenes (OPVs),^4^ oligophenyleneethynylenes (OPEs),^5^ oligoanilines (OAs)^6^ and oligothiophenes.^7^ Among them, OPEs have been particularly employed because of their excellent photophysical and electronic properties, which have been exploited for many interesting applications, such as light emitting diodes (LEDs),^8^ field-effect transistors (FETs),^9^ organic photovoltaic devices,^10^ sensing,^11^ imaging^12^ and thermoelectric materials.^13^ As for many other classes of π-conjugated systems, the optical properties of OPEs can be finely tuned by chemical functionalization.^14^

In addition to their well-known functional properties, efforts were also taken to modulate the self-assembly behaviour of OPEs in solution, owing to the importance of supramolecular structures of these systems in organic electronics.^15^ However, an inherent disadvantage of the oligomeric OPE backbone, at least compared to other types of dyes, is its comparatively smaller spectroscopic changes during self-assembly,^16^ which makes detailed supramolecular investigations challenging. This can be explained by the smaller, less polar and more conformationally flexible carbon-only aromatic surface of the OPEs, which decreases the association constant compared to typical dyes with fused aromatic rings, heteroatoms or polar substituents.^17^ For this reason, additional functional groups for hydrogen bonding are typically attached to the OPEs core in order to enhance their aggregation propensity and, in turn, facilitate their supramolecular analysis by spectroscopy.^16,18^

However, despite numerous reports on OPE-based hydrogen-bonded assemblies, it is surprising that no systematic studies are available for OPEs where aromatic interactions are the chief driving force for aggregation. To bridge this knowledge gap, we herein unravel the effect of π-conjugation length on the optical and self-assembly behaviour of OPEs that are primarily governed by aromatic interactions. Our studies allow to unveil the relative contribution of the OPE core to supramolecular polymerization by establishing a structure-self-assembly relationship. Accordingly, we designed and synthesized linear OPEs with different π–length (**OPE3–7**) to compare the photophysical and self-assembly behaviour in solution (Scheme 1).

**Scheme 1:**
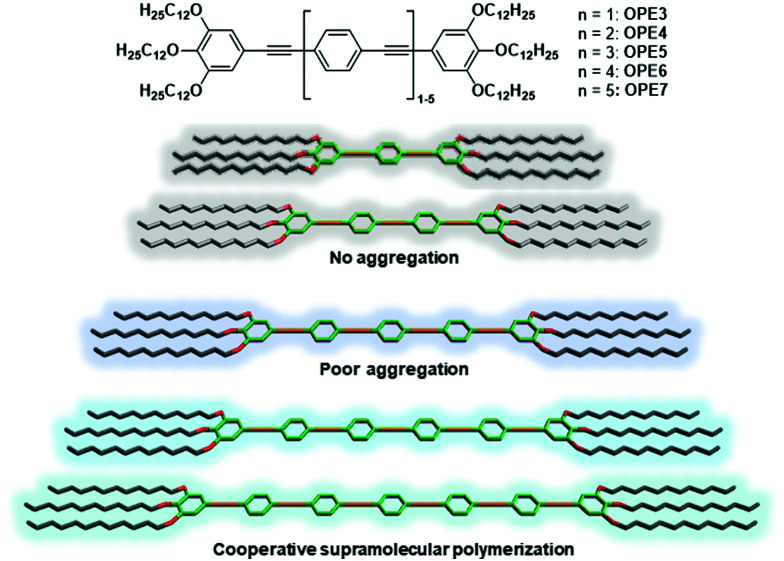
Chemical structure of **OPE3–7** (top) and classification of their self-assembly behaviour in solution depending on the conjugation length (bottom).

The synthesis of **OPE3–7** was accomplished using multi-step synthetic protocols based on previous reports as described in the electronic supplementary information (ESI†). All molecules have been characterized using spectroscopic techniques (^1^H and ^13^C NMR, FTIR, HRMS (ESI†)).

Solvent-dependent UV-Vis and fluorescence studies allowed us to identify chloroform (CHCl_3_) and methylcyclohexane (MCH) as the good and poor solvents, respectively (Fig. S1–S5, ESI†). Based on that, the effect of conjugation length on the photophysical properties of the OPEs was monitored by UV-Vis and emission spectroscopy dissolving **OPE3–7** in CHCl_3_ (*c* = 1 × 10^−5^ M). As expected, increasing the conjugation length from **OPE3** to **OPE6** induces a progressive red shift in the absorption maximum (from 337 to 363 nm) and a concomitant increase in the molar absorption coefficient (*ε*) (Fig. 1a). **OPE7** also follows this trend in terms of red shifted absorption compared to **OPE6** (Fig. 1a). However, unexpectedly, the absorption intensity of **OPE7** is decreased compared to that of **OPE6**, and a minor shoulder band at longer wavelengths is also noticeable. These results can be ascribed to a weak aggregation of **OPE7** because of its larger π–surface compared to **OPE6**, leading to the observed weakened absorption. Emission studies also showed a red shift in the emission maximum (410 to 428 nm) upon increasing conjugation length from **OPE3** to **OPE5** (Fig. 1b). This is accompanied by a gradual decrease in the emission intensity (Fig. 1b), possibly due to the higher rotational degrees of freedom upon increasing the number of phenylethynyl units.^19^ Further increase in the π–length results in negligible shifts in the emission maximum; however, an additional shoulder band becomes apparent for **OPE6–7** at *ca.* 410 nm (Fig. 1c), which might be the result of short oligomer formation. This solvent- and conjugation length-dependent absorption and emission properties are also visible to the naked eye, as shown in the photographs of the corresponding solutions (Fig. 1d).

**Fig. 1:**
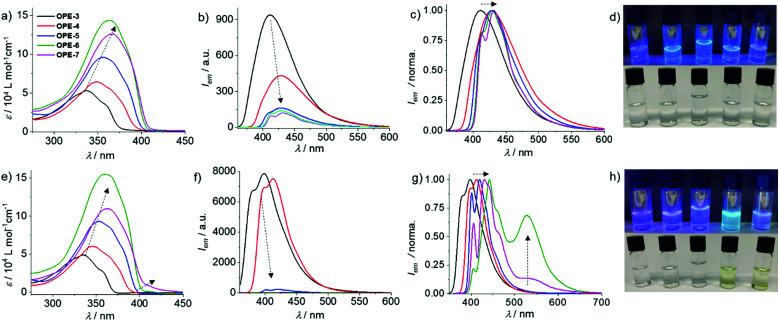
(a and e) UV-Vis, (b and f) fluorescence (*λ*_exc_ = 335–365 nm), (c and g) normalized emission spectra and (d and h) photographs under UV and daylight of **OPE3–7** in CHCl_3_ (a–d) and MCH (e–h) (*c* = 1 × 10^−5^ M, 298 K).

Subsequently, we examined the potential aggregation behaviour of **OPE3–7** in MCH. In analogy to the UV-Vis spectra in CHCl_3_, a simultaneous red shift and increase in absorption was observed when moving from **OPE3** to **OPE5** (Fig. 1e), indicating no to poor aggregation in MCH. On the other hand, **OPE6** and **OPE7** showed more significant changes, particularly **OPE7**. For this derivative, a broad shoulder band at *ca.* 410 nm that spreads up to *ca.* 450 nm appears, which points to aggregation in MCH. Therefore, as expected, increasing the conjugation length enhances the aggregation tendency *via* aromatic interactions. Further, emission studies for **OPE3–7** in MCH were performed under identical experimental conditions. For **OPE3–4**, a strong and structured emission was observed, indicating a molecularly dissolved state (Fig. 1f). Notably, moving from **OPE4** to **OPE5** leads to a significant drop in emission intensity, suggesting the initial stages of an aggregation event for **OPE5**, which does not occur for **OPE3,4** under the same experimental conditions. Unsurprisingly, further increasing the π–length to **OPE6** and **OPE7** causes a further emission quenching due to stronger aggregation (Fig. 1f). This phenomenon is further characterized by the appearance of an additional shoulder for **OPE6–7** at *ca.* 540 nm in emission experiments (Fig. 1g). The absence of such shoulder band for **OPE5** might be due to only partial or incomplete aggregation in MCH under these conditions. The proposed aggregation for **OPE6** and **OPE7** in MCH under the selected experimental conditions is also visible by the naked eye, as only the MCH solutions of these two derivatives show a light-yellow color whereas the solutions of **OPE3–5** are colorless (Fig. 1h). In accordance with the appreciable luminescence observed in Fig. 1h, **OPE3–7** exhibited fluorescence quantum yields (*Φ*_F_) of 40–50% in MCH and CHCl_3_ (Table S1, ESI†).

Subsequently, we conducted concentration-dependent UV-Vis and fluorescence studies to further examine the self-assembly. As expected, **OPE3–5** display no significant changes in the absorption spectra upon increasing concentration from 5 × 10^−5^ M to 1 × 10^−3^ M (Fig. S6–S8, ESI†). These results support the previous hypothesis that these derivatives exhibit only poor aggregation in MCH, presumably due to the shorter OPE core. In contrast, **OPE6** showed clear signs of aggregation in MCH upon increasing the concentration from 2.5 × 10^−6^ M to 100 × 10^−6^ M, as evident from the slight red shift in the absorption maximum and the emergence of a shoulder band at around 410 nm (Fig. 2a and Fig. S9a, ESI†). Similarly, a remarkable red shift in emission (440 nm to 530 nm) was observed for **OPE6** in MCH upon increasing the concentration from 7.5 × 10^−6^ M to 1 × 10^−3^ M (Fig. S9b and c, ESI†). The changes in the absorption and emission studies upon increasing concentration suggest both a slipped and twisted arrangement of the monomers during aggregation.^20^ Finally, in analogy to **OPE6**, **OPE7** displays a red shifted shoulder when the concentration is increased from 5 × 10^−6^ M to 500 × 10^−6^ M, diagnostic of aggregate formation (Fig. 2b and Fig. S10a, ESI†). Notice that above 1 × 10^−4^ M the aggregate band becomes sharp, which can be due to the precise organization of **OPE7** with largest π-system. Additionally, a red shift in the emission was also observed for **OPE7** with increasing concentration, indicating a similar aggregation mode as that observed for **OPE6** (Fig. S10b, ESI†). However, in contrast to **OPE6**, **OPE7** precipitates over time above 1 × 10^−4^ M, which is concomitant with a drop in fluorescence, as evident in the photographs shown in Fig. S10c (ESI†).

**Fig. 2:**
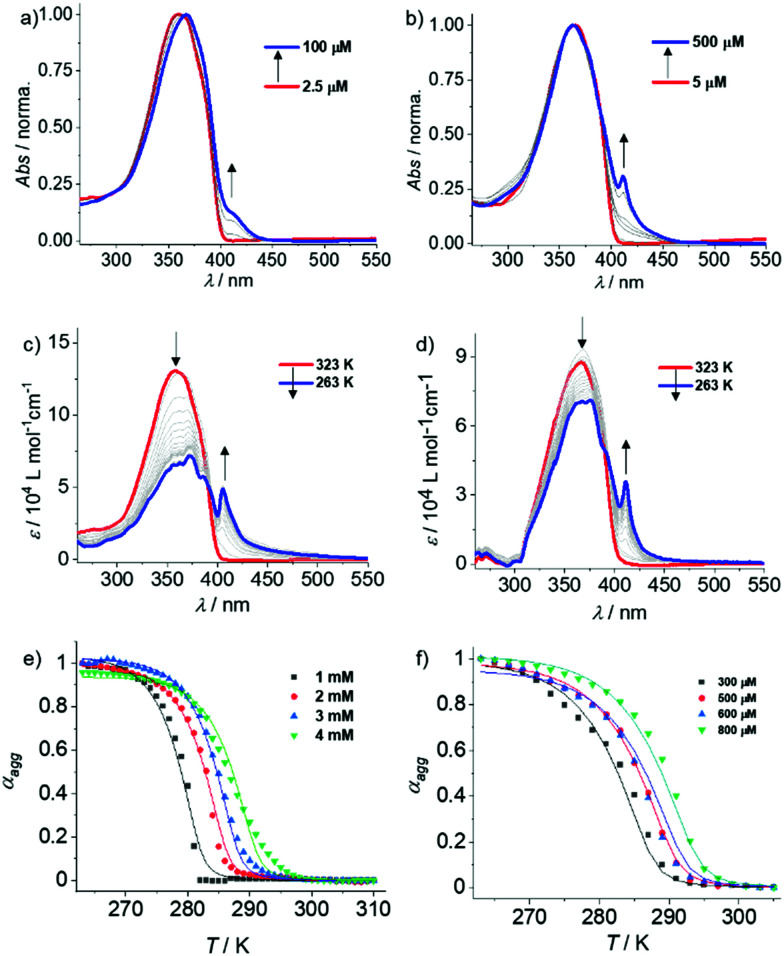
Concentration-dependent UV-Vis spectra of (a) **OPE6** and (b) **OPE7** in MCH at 298 K. Variable temperature (VT)-UV-Vis spectra of (c) **OPE6** (*c* = 1 × 10^−3^ M) and (d) **OPE7** (*c* = 3 × 10^−4^ M) in MCH. Global fitting of the cooling curves derived from the corresponding VT experiments of (e) **OPE6** (*c* = 1 × 10^−3^–4 × 10^−3^ M, *λ* = 405 nm) and (f) **OPE7** (*c* = 3 × 10^−4^–8 × 10^−4^ M, *λ* = 411 nm).

Further, we performed variable temperature (VT)-UV-Vis experiments for **OPE3–7** in MCH to elucidate the thermodynamics of their supramolecular polymerization. As expected, no changes in the UV-Vis spectrum were observed for **OPE3** and **OPE4** (*c* = 1 × 10^−3^ M) upon varying temperature from 293 K to 263 K (Fig. S11, ESI†). On the other hand, a noticeable broadening of the UV-Vis spectra was observed for **OPE5** in MCH under similar experimental conditions, however with no shoulder band formation (Fig. S12a, ESI†). These findings suggest the initial stages of an aggregation event, which may also provide a plausible explanation for the reduced emission intensity of **OPE5** compared to **OPE3,4** (*vide supra*). Interestingly, raising the concentration of **OPE5** to 2 × 10^−3^ M and further to 5 × 10^−3^ M (where spectral saturation is rather significant) allowed us to identify the red shifted shoulder band at *ca.* 410 nm that was also characteristic for the aggregates of **OPE6** and **OPE7** (Fig. S12c and d, ESI†). However, due to the high concentrations required to assemble **OPE5** (above *ca.* 4 mM), a quantitative thermodynamic analysis of this derivative is not possible. VT-^1^H-NMR experiments were also performed for **OPE3–5** in MCH-*d*_14_ (*c* = 5 mM, Fig. S13, ESI†), which, unlike **OPE6,7**, are sufficiently soluble for these studies. In agreement with previous observations, the π–stacking tendency increases in the order **OPE3** → **OPE4** → **OPE5**, as evident from the more significant broadening of the aromatic signals at low temperatures (263 K–253 K) upon increasing the π–length.

We next examined the self-assembly behaviour of **OPE6** by VT-UV-Vis. Notably, upon cooling a hot solution of **OPE6** (*c* = 1 × 10^−3^ M) in MCH from 323 K to 263 K, the monomeric band at 360 nm decreases at the expense of the previously described sharp aggregate band at 410 nm (Fig. 2c). Similarly, VT-UV-Vis cooling experiments of **OPE7** in MCH also lead to aggregation (Fig. 2d); however, lower concentrations are required (*c* = 3 × 10^−4^ M) due to its higher aggregation propensity resulting from the larger π–surface. Distinct UV-Vis spectral changes corresponding to aggregate formation were also observed for **OPE6** and **OPE7** in VT-UV-Vis at different concentrations (Fig. S14 and S15, ESI,† respectively). The plots of *α*_agg_*vs.* temperature extracted from these experiments were globally fitted to the cooperative (nucleation–elongation) model (Fig. 2e and f).^21^ Both derivatives show a comparable, yet relatively low degree of cooperativity, which can be explained by the absence of additional non-covalent interactions such as hydrogen bonding.^22^ This effect also explains the low values of elongation constant (*K*_e_) (in the range of 10^2^–10^3^ M^−1^) as well as low negative Δ*G*° values (−8.62 and −16.56 kJ mol^−1^ for **OPE6** and **OPE7**, respectively; Tables S2 and S3, ESI†). The additional stability of the **OPE7** aggregates compared to **OPE6** can be ascribed to the increase in conjugation length.

Ultimately, atomic force microscopy (AFM) for both aggregates **OPE6** and **OPE7** at 2.5 × 10^−4^ M on mica allowed us to visualize entangled networks of fibers with 3–5 nm height and 200–300 nm width (Fig. 3). The fact that the heights are considerably smaller than the molecular length of **OPE6,7** suggests a tilted and possibly slipped stacking of the OPEs on the substrate. Scanning Electron Microscopy (SEM) on silicon wafer further confirmed the fiber formation of **OPE6** and **OPE7** (Fig. S18, ESI†).

**Fig. 3:**
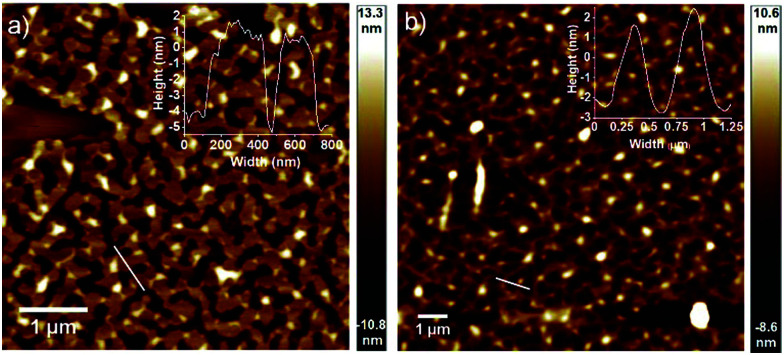
Height AFM images of (a) **OPE6** and (b) **OPE7** (*c* = 2.5 × 10^−4^ M) on mica.

In conclusion, we have unravelled the relationship between conjugation length and self-assembly behaviour in linear OPEs. Spectroscopic studies demonstrate a red shift both in absorption and emission upon increasing the conjugation length. The aggregation propensity in nonpolar media also follows this trend (**OPE3** → **OPE7**). At mM concentrations, a minimum of five aromatic rings (**OPE5**) is required to drive aggregation *via* aromatic interactions. This is most pronounced for the largest derivatives of the series (**OPE6,7**), leading to emissive fiber-like aggregates. Although the self-assembly mainly occurs by aromatic and solvophobic interactions, both derivatives follow the cooperative mechanism. These results contrast with most self-assemblies that are mainly stabilized by aromatic interactions (without hydrogen bonding), indicating that possible planarization events of the conformationally unrestricted OPE core can turn on cooperative effects. Our studies may contribute to a better understanding of the structure/property relationship in π-conjugated systems.

We thank the Alexander Humboldt foundation (Sofja Kovalevskaja Award), the Deutsche Forschungsgemeinschaft (SFB858) and the European Research Council (ERC-StG-2016 SUPRACOP-715923) for funding. We thank Jonas Matern for the SEM measurements.

## Conflicts of interest

There are no conflicts to declare.

## Supplementary Material

CC-057-D1CC01054A-s001

## References

[cit1] Babu S. S., Praveen V. K., Ajayaghosh A. (2014). Chem. Rev..

[cit2] Babu S. S., Kartha K. K., Ajayaghosh A. (2010). J. Phys. Chem. Lett..

[cit3] Shen B., Kim Y., Lee M. (2020). Adv. Mater..

[cit4] Praveen V. K., Ranjith C., Bandini E., Ajayaghosh A., Armaroli N. (2014). Chem. Soc. Rev..

[cit5] Albert S. K., Thelu H. V. P., Golla M., Krishnan N., Chaudhary S., Varghese R. (2014). Angew. Chem., Int. Ed..

[cit6] Zhou C. Q., Ren Y. Y., Han J., Gong X. X., Wei Z. X., Xie J., Guo R. (2018). J. Am. Chem. Soc..

[cit7] Tovar J. D. (2013). Acc. Chem. Res..

[cit8] Yamaguchi Y., Ochi T., Matsubara Y., Yoshida Z. (2015). J. Phys. Chem. A.

[cit9] Schmid S., Koser S., Melzer C., Mankel E., Bunz U. H. F. (2018). Synth. Met..

[cit10] Nierengarten J.-F., Gu T., Aernouts T., Geens W., Poortmans J., Hadziioannou G., Tsamouras D. (2004). Appl. Phys. A: Mater. Sci. Process..

[cit11] Tang Y., Achyuthan K. E., Whitten D. G. (2010). Langmuir.

[cit12] Thirumalai R., Mukhopadhyay R. D., Praveen V. K., Ajayaghosh A. (2015). Sci. Rep..

[cit13] Sadeghi H. (2019). J. Phys. Chem. C.

[cit14] Roy S., Samanta D., Bhattacharyya S., Kumar P., Hazra A., Maji T. K. (2018). J. Phys. Chem. C.

[cit15] Fernández G., García F., Aparicio F., Matesanz E., Sánchez L. (2009). Chem. Commun..

[cit16] Philips D. S., Kartha K. K., Politi A. T., Krüger T., Albuquerque R. Q., Fernández G. (2019). Angew. Chem., Int. Ed..

[cit17] Chen Z., Lohr A., Saha-Möller C. R., Würthner F. (2009). Chem. Soc. Rev..

[cit18] Gopal A., Varghese R., Ajayaghosh A. (2012). Chem. – Asian J..

[cit19] Levitus M., Schmieder K., Ricks H., Shimizu K. D., Bunz U. H. F., Garcia-Garibay M. A. (2001). J. Am. Chem. Soc..

[cit20] Dünnebacke T., Kartha K. K., Wiest J. M., Albuquerque R. Q., Fernández G. (2020). Chem. Sci..

[cit21] Ten Eikelder H. M. M., Markvoort A. J., De Greef T. F. A., Hilbers P. A. J. (2012). J. Phys. Chem. B.

[cit22] Rest C., Kandanelli R., Fernández G. (2015). Chem. Soc. Rev..

