# Supplementary material for: Understanding the role of conjugation length on the self-assembly behaviour of oligophenyleneethynylenes
Source: Chem Commun (Camb). 2021 Apr 27;57(40):4890–3. doi: 10.1039/d1cc01054a (PMC8132183; doi:10.1039/d1cc01054a)
Supplement: CC-057-D1CC01054A-s001 [file CC-057-D1CC01054A-s001.pdf]

Electronic Supplementary Material (ESI) for ChemComm.
This journal is © The Royal Society of Chemistry 2021

Supporting Information for

Understanding the role of conjugation length on the self-assembly behaviour of oligophenyleneethynylenes

Beatriz Matarranz,[a] Goutam Ghosh,[a] Ramesh Kandanelli,[b] Angel Sampedro,[a] Kalathil K. Kartha*[a] and Gustavo Fernández*[a]

[a]Organisch-Chemisches Institut, Universität Münster, Corrensstraße 36, 48149 Münster, Germany

[b]Institut für Organische Chemie, Universität Würzburg am Hubland, 97074 Würzburg, Germany

Table of Contents

1 Solvent-dependent spectroscopic analysis 3

2 Concentration-dependent spectroscopic analysis 5

3 Temperature-dependent spectroscopic analysis 9

3.1 Nucleation-Elongation Model for Cooperative Supramolecular Polymerizations 12

3.2 Thermodynamic Parameters 13

4 Microscopic analysis of OPE6 and OPE7 13

5 Experimental Part 16

5.1 Materials and Methods 16

5.2 Synthesis and Characterization of OPEs 3-7 17

5.3 ^{1}H and ^{13}C NMR Spectra 20

6 References 24

1 Solvent-dependent spectroscopic analysis

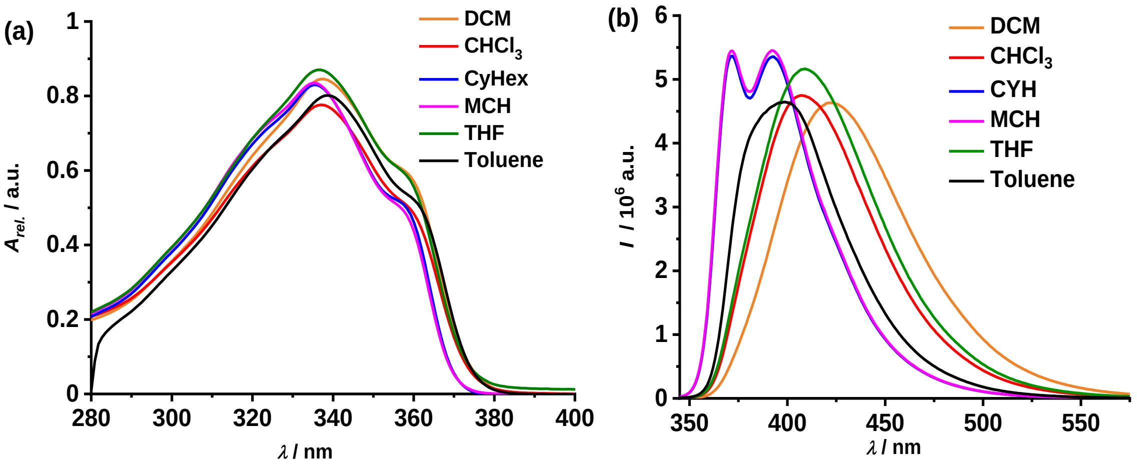

Figure S1. Solvent-dependent a) UV/Vis (c = 1 x 10^{-4} M) and b) fluorescence (c = 1 x 10^{-5} M, λ_{exc} = 335 nm) spectra of **OPE3**.

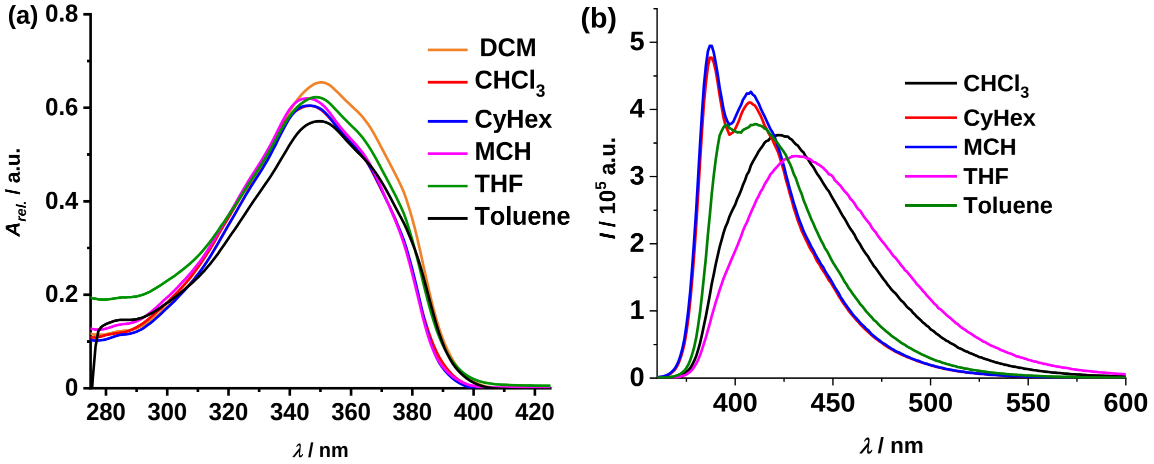

Figure S2. Solvent-dependent a) UV/Vis (c = 1 x 10^{-4} M) and b) fluorescence (c = 1 x 10^{-5} M, λ_{exc} = 345 nm) spectra of **OPE4**.

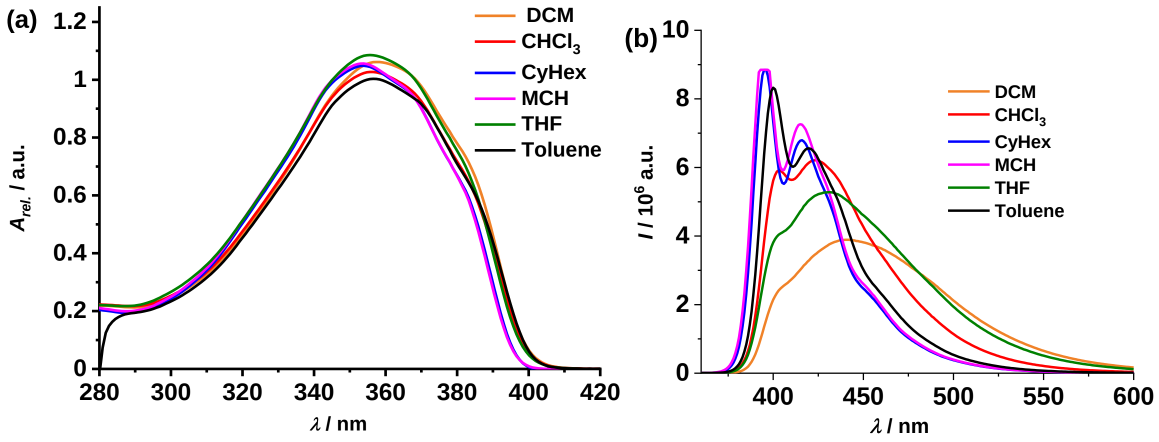

Figure S3. Solvent-dependent a) UV/Vis (c = 1 x 10^{-4} M) and b) fluorescence (c = 1 x 10^{-5} M, λ_{exc} = 350 nm) spectra of **OPE5**.

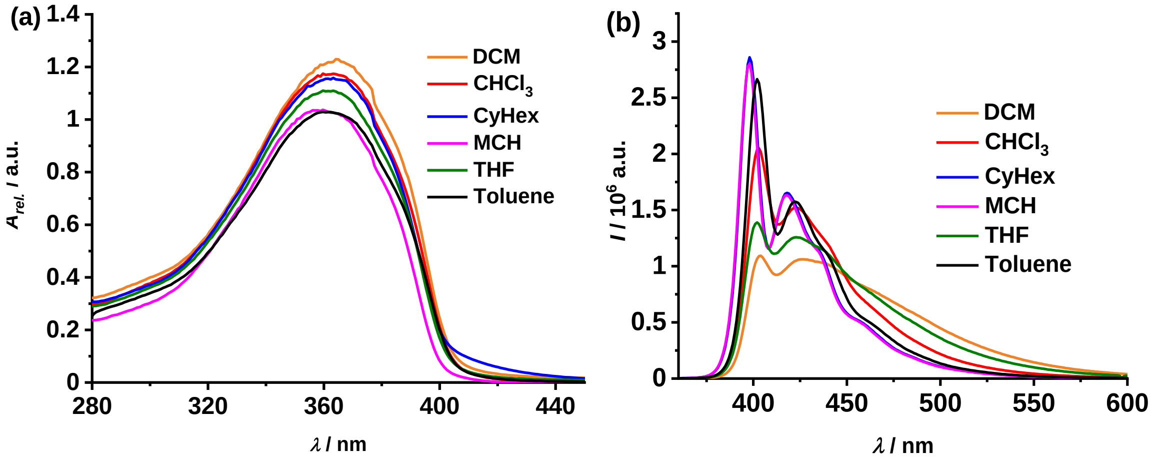

Figure S4. Solvent-dependent a) UV/Vis (c = 1 x 10^{-4} M) and b) fluorescence (c = 1 x 10^{-5} M, λ_{exc} = 355 nm) spectra of **OPE6**.

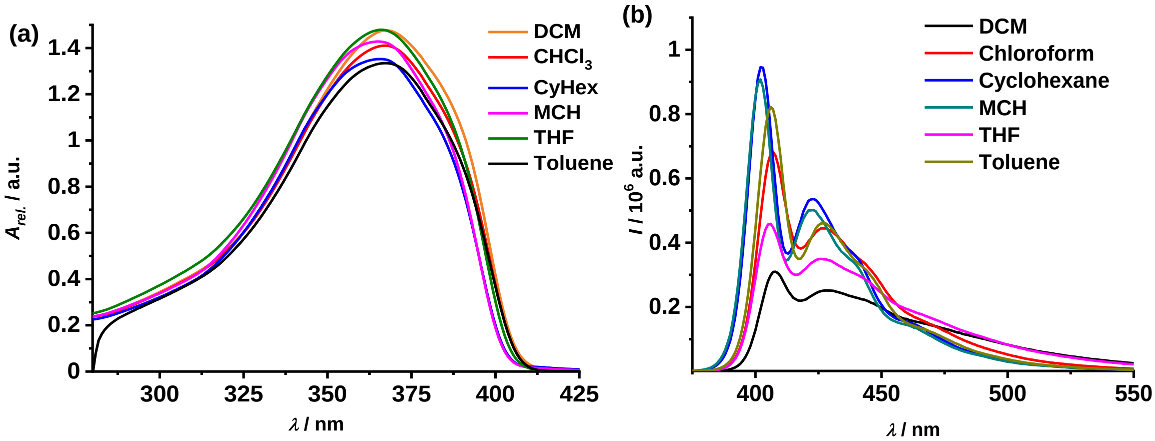

Figure S5. Solvent-dependent a) UV/Vis (c = 1 x 10^{-5} M) and b) fluorescence (c = 1 x 10^{-5} M, λ_{exc} = 365 nm) spectra of **OPE7**.

Table S1. Fluorescence Quantum Yield values for the different OPEs in $CHCl_3$ and MCH. Anthracene in EtOH (Φ_F: 0.28, λ_{exc}: 340nm) was used as reference for the determination of the Φ_F.

	**OPE3**	**OPE4**	**OPE5**	**OPE6**	**OPE7**
**$CHCl_3$**	0.47	0.44	0.40	0.52	0.46
**MCH**	0.43	0.48	0.46	0.47	0.46

2 Concentration-dependent spectroscopic analysis

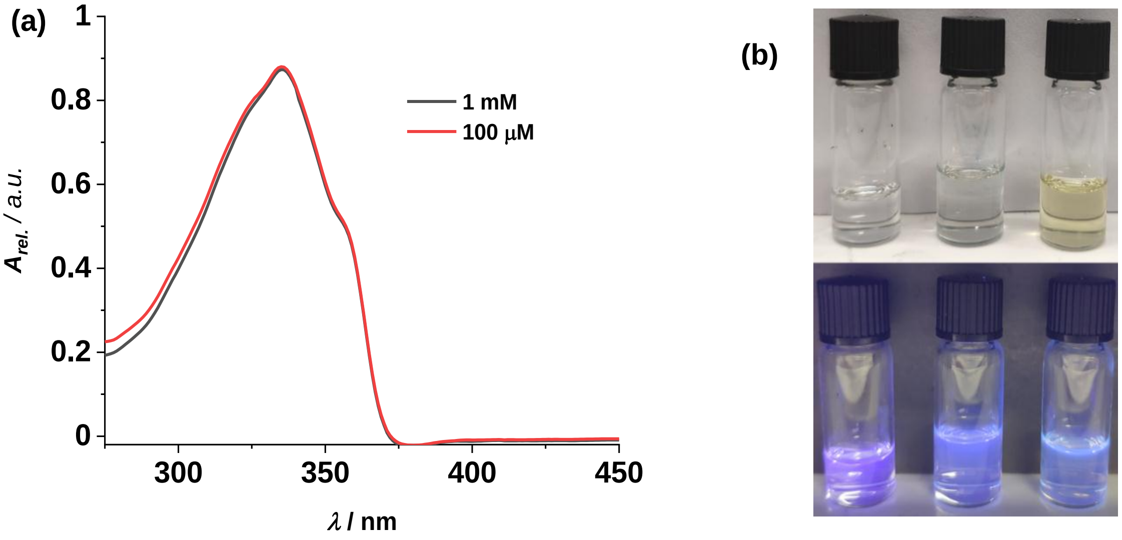

Figure S6. a) Concentration-dependent UV-Vis spectra of **OPE3** (MCH, 298 K) and b) photographs of **OPE3** solutions in MCH at 50 µM (left), 100 µM (middle) and 1 mM (right) under daylight (top) and 365 nm UV light (bottom).

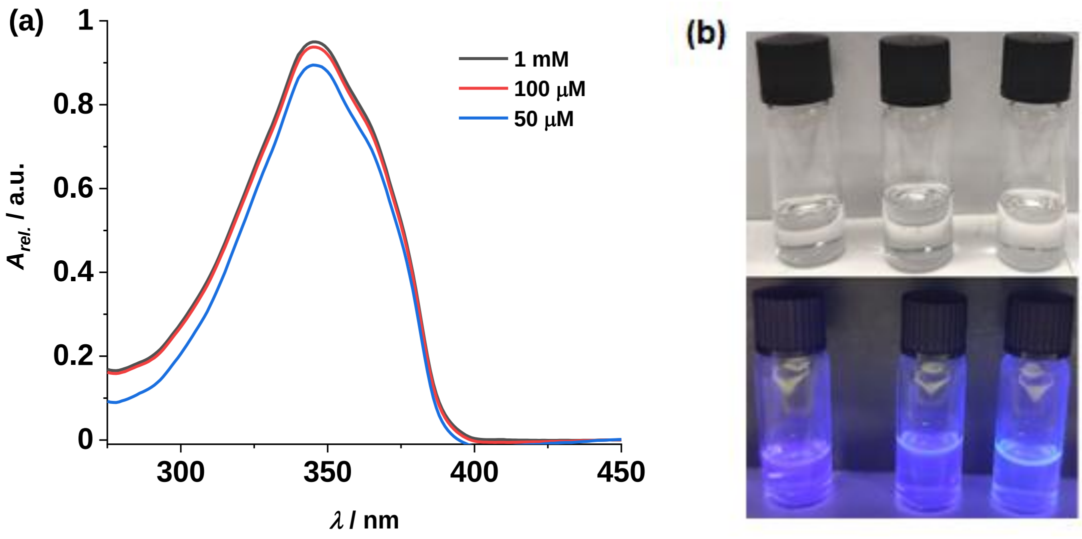

Figure S7. a) Concentration-dependent UV-Vis spectra of **OPE4** (MCH, 298 K) and b) photographs of **OPE4** solutions in MCH at 50 µM (left), 100 µM (middle) and 1 mM (right) under daylight (top) and 365 nm UV light (bottom).

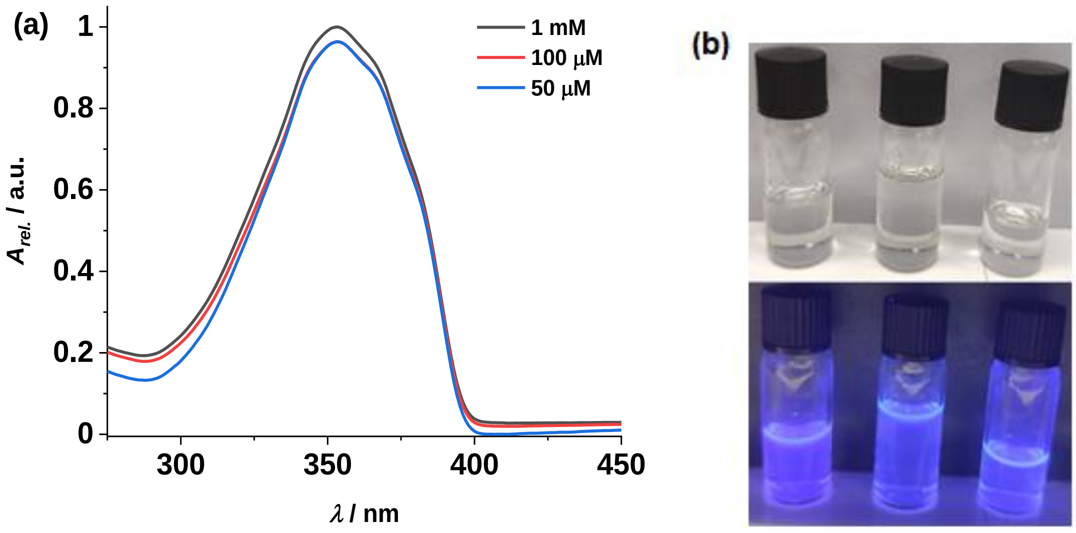

Figure S8. a) Concentration-dependent UV-Vis spectra of **OPE5** (MCH, 298 K) and b) photographs of **OPE5** solutions in MCH at 50 μM (left), 100 μM (middle) and 1 mM (right) under daylight (top) and 365 nm UV light (bottom).

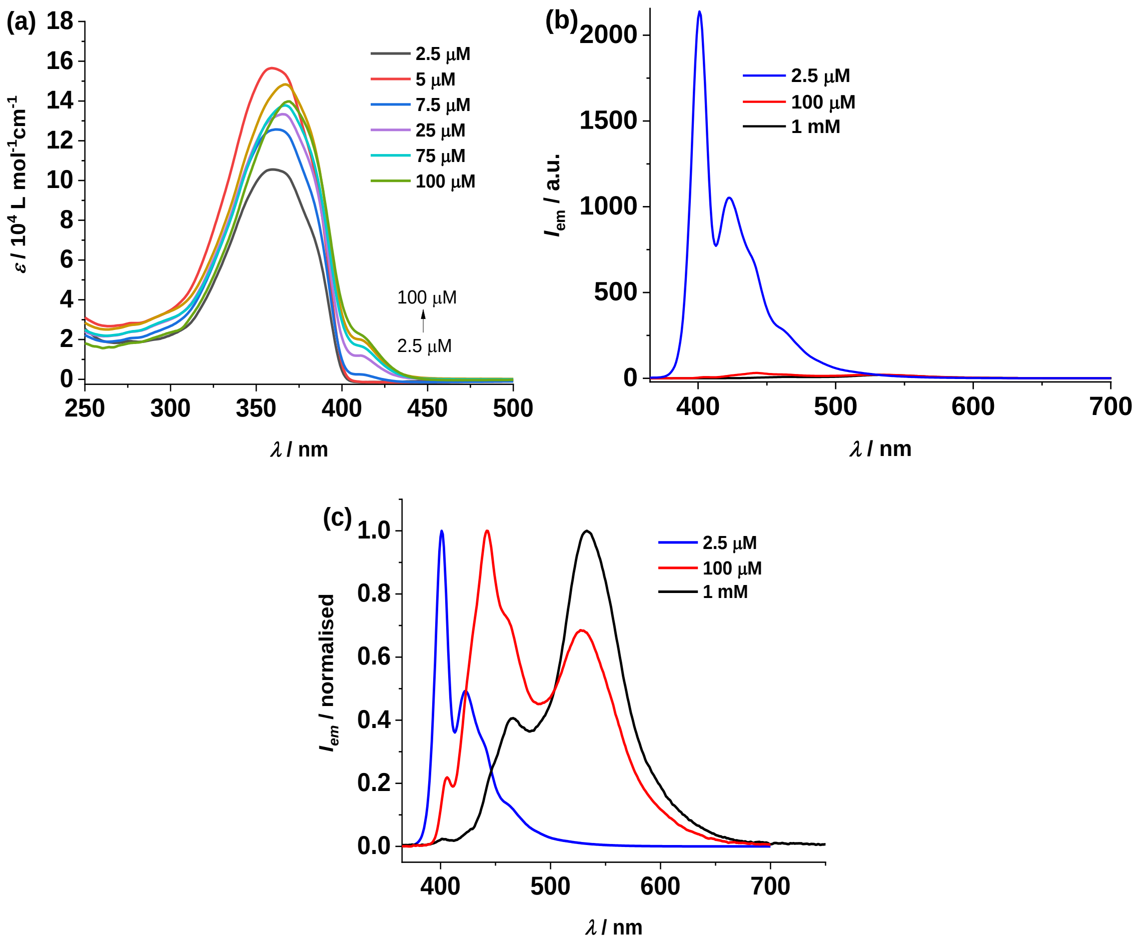

(d)

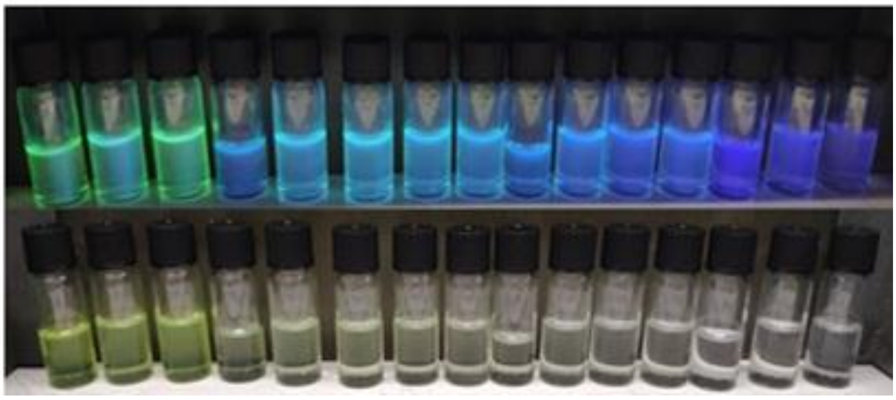

Figure S9. Concentration-dependent a) UV/Vis, b) fluorescence and c) corresponding normalised fluorescence spectra of **OPE6** (MCH, 298 K, $\lambda_{exc.}$ = 355 nm). d) Photographs of **OPE6** solutions in MCH at different concentrations from 1 mM (left) to 2.5 μM (right) under 365 nm UV light (top) and daylight (bottom).

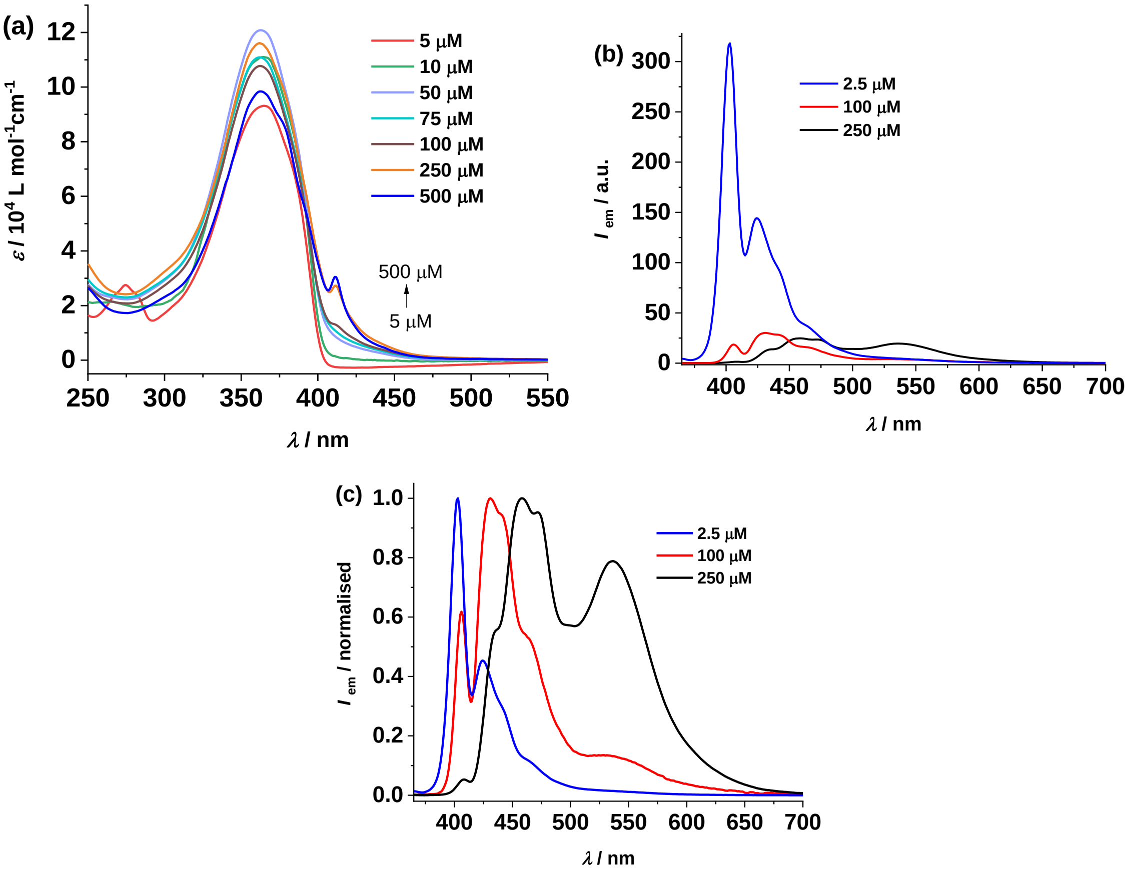

(d)

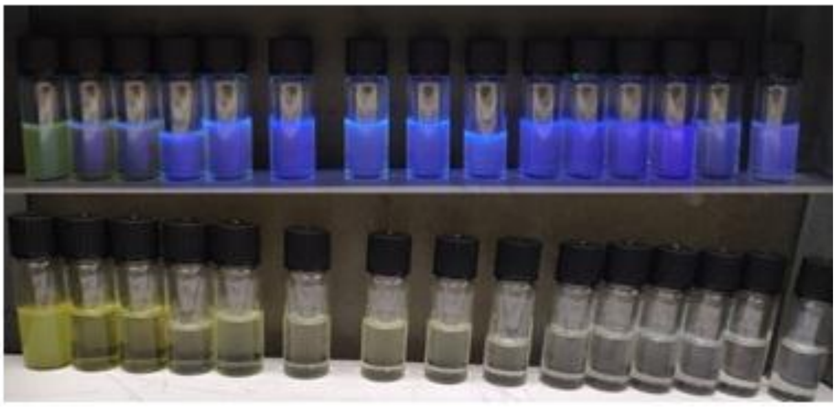

Figure S10. Concentration-dependent a) UV/Vis, b) fluorescence and c) corresponding normalised fluorescence spectra of **OPE7** (MCH, 298 K, $\lambda_{exc.}$ = 355 nm). d) Photographs of **OPE7** solutions in MCH at different concentrations from 1 mM (left) to 2.5 µM (right) under 365 nm UV light (top) and daylight (bottom).

Comparing the emission properties of **OPE6** and **OPE7** in the aggregated state in MCH, it can be noticed that the emission band at ca. 530 nm is more significant for **OPE6** than it is for **OPE7** (see Figures S9 vs. S10). A plausible hypothesis is an increased population of coplanar OPEs for **OPE6** compared to **OPE7**. Experimentally, we also observed a much lower solubility of **OPE7** in MCH compared to **OPE6**, which leads to the formation of cloudy or partially precipitated solutions for **OPE7** (such as displayed in Figure S10d) upon increasing concentration. This is not the case for **OPE6** (see Figure S9d), where all solutions (even at mM concentration) remain transparent. On this basis, we hypothesize that the interplay between solvation effects and the different population of twisted and co-planar OPEs for **OPE6** and **OPE7** might account for the differences in photophysical behavior.

3 Temperature-dependent spectroscopic analysis

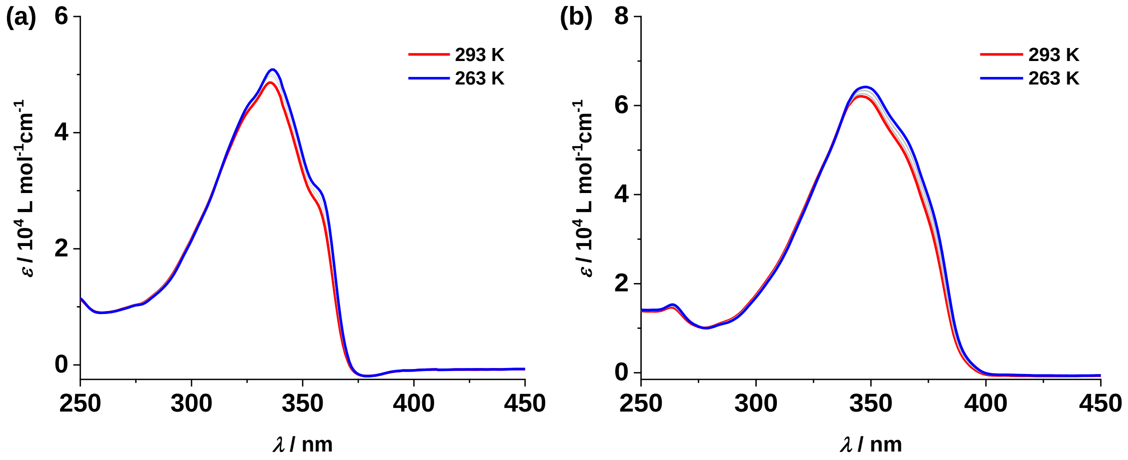

Figure S11. Variable temperature (VT)-UV/Vis spectra of a) **OPE3** and b) **OPE4** in MCH from 293 K to 263 K (c = 1 x 10^{-3} M, cooling rate: 1 K/min)

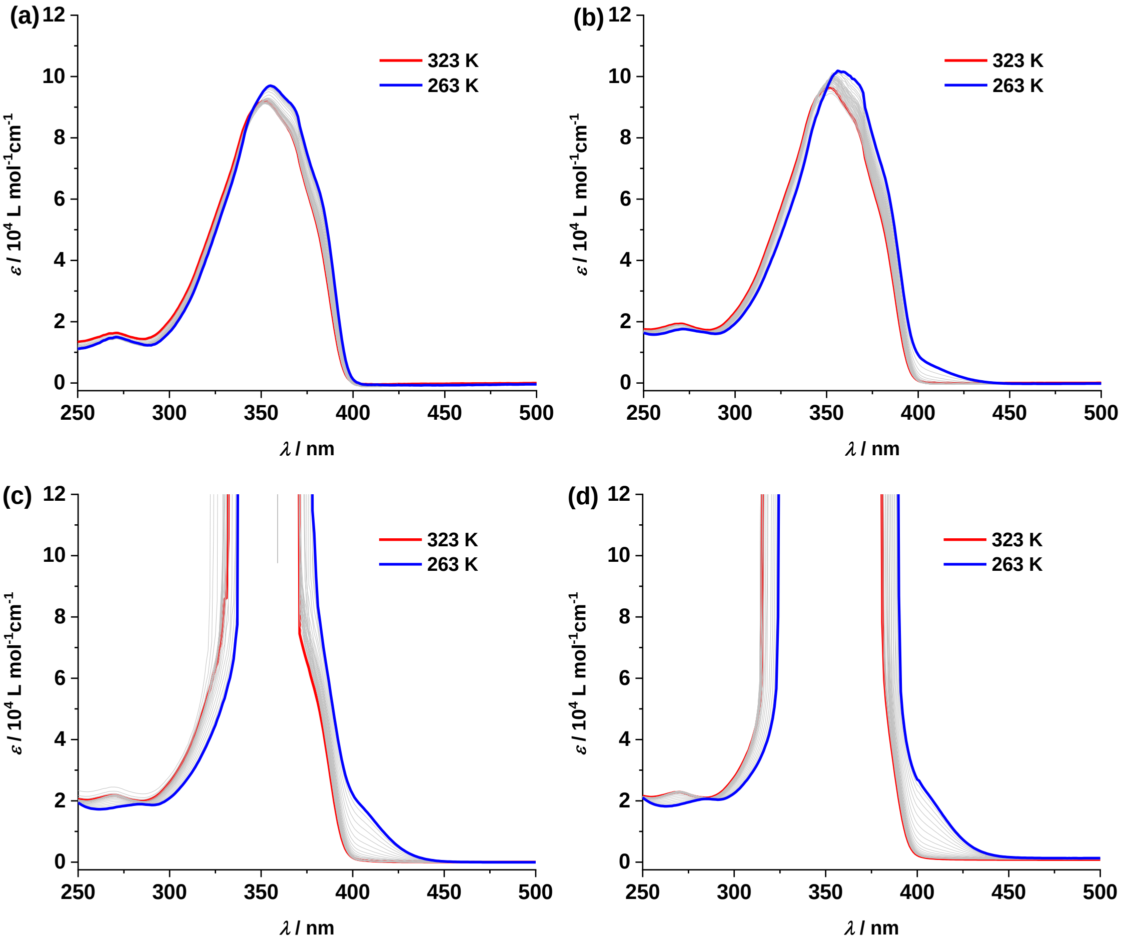

Figure S12. VT-UV/Vis spectra of **OPE5** in MCH from 323 K to 263 K, with a cooling rate of 1 K/min at a) 1 mM, b) 2 mM, c) 4 mM and d) 5 mM concentration.

a)

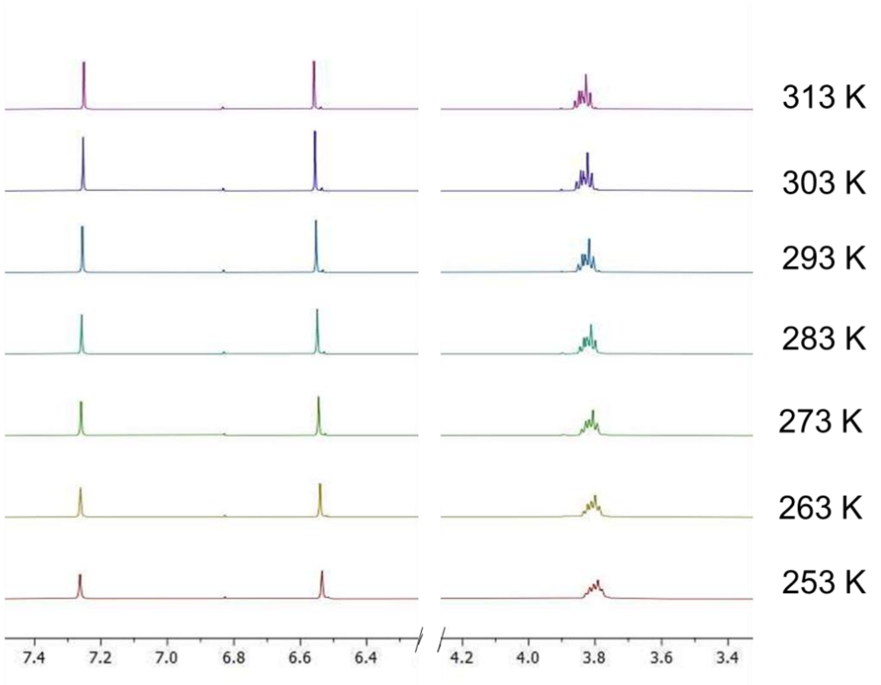
313 K
303 K
293 K
283 K
273 K
263 K
253 K
7.4
7.2
7.0
6.8
6.6
6.4
4.2
4.0
3.8
3.6
3.4

b)

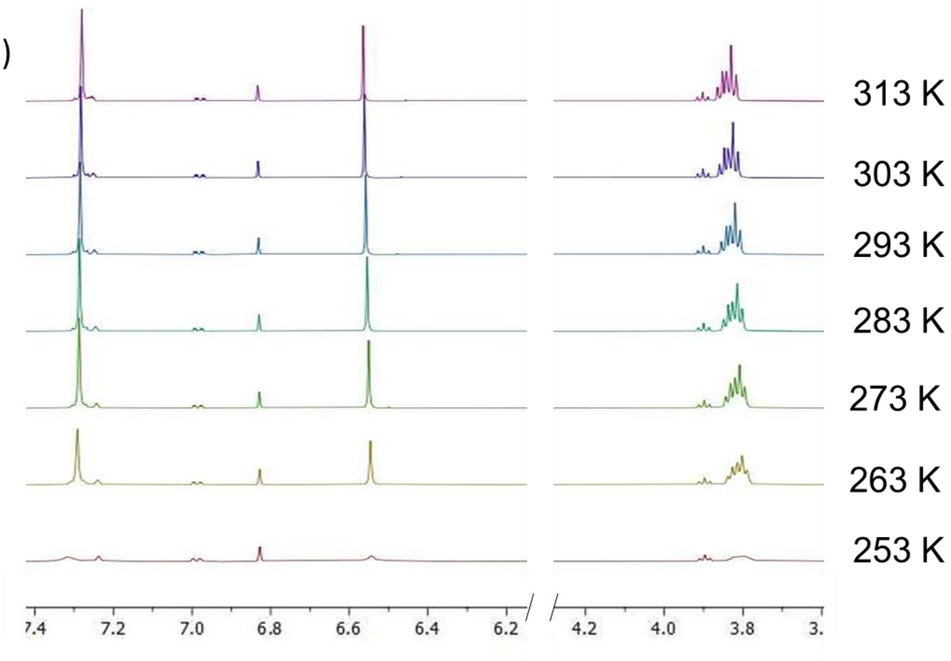
313 K
303 K
293 K
283 K
273 K
263 K
253 K
7.2
7.0
6.8
6.6
6.4
6.2
4.2
4.0
3.8

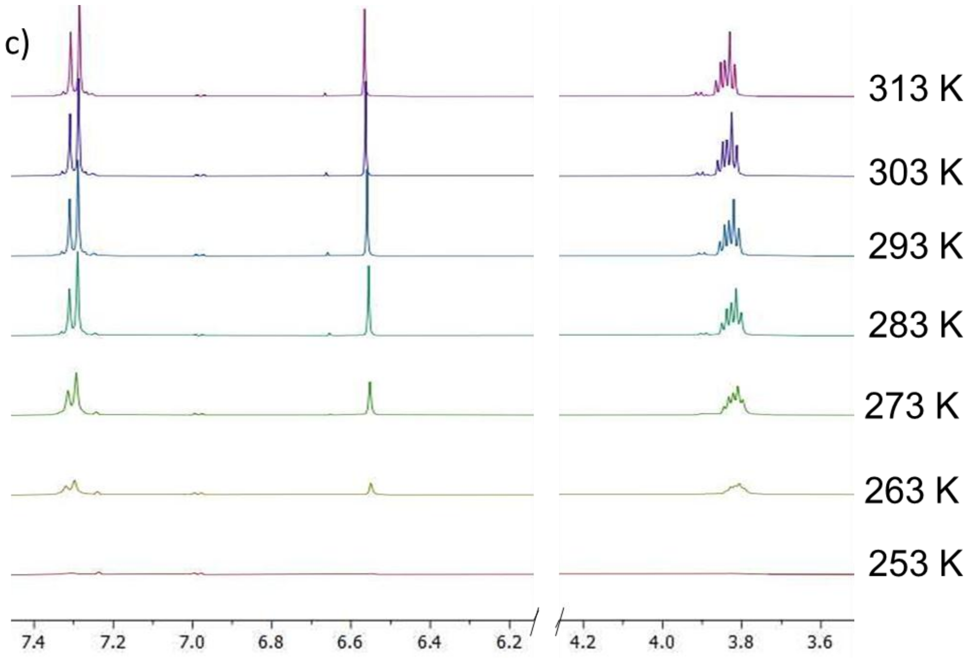

Figure S13. VT-^{1}H-NMR spectra of a) **OPE3**, b) **OPE**4 and c) **OPE5** in MCH-d_{14} between 313 K and 253 K with a concentration of 5 mM.

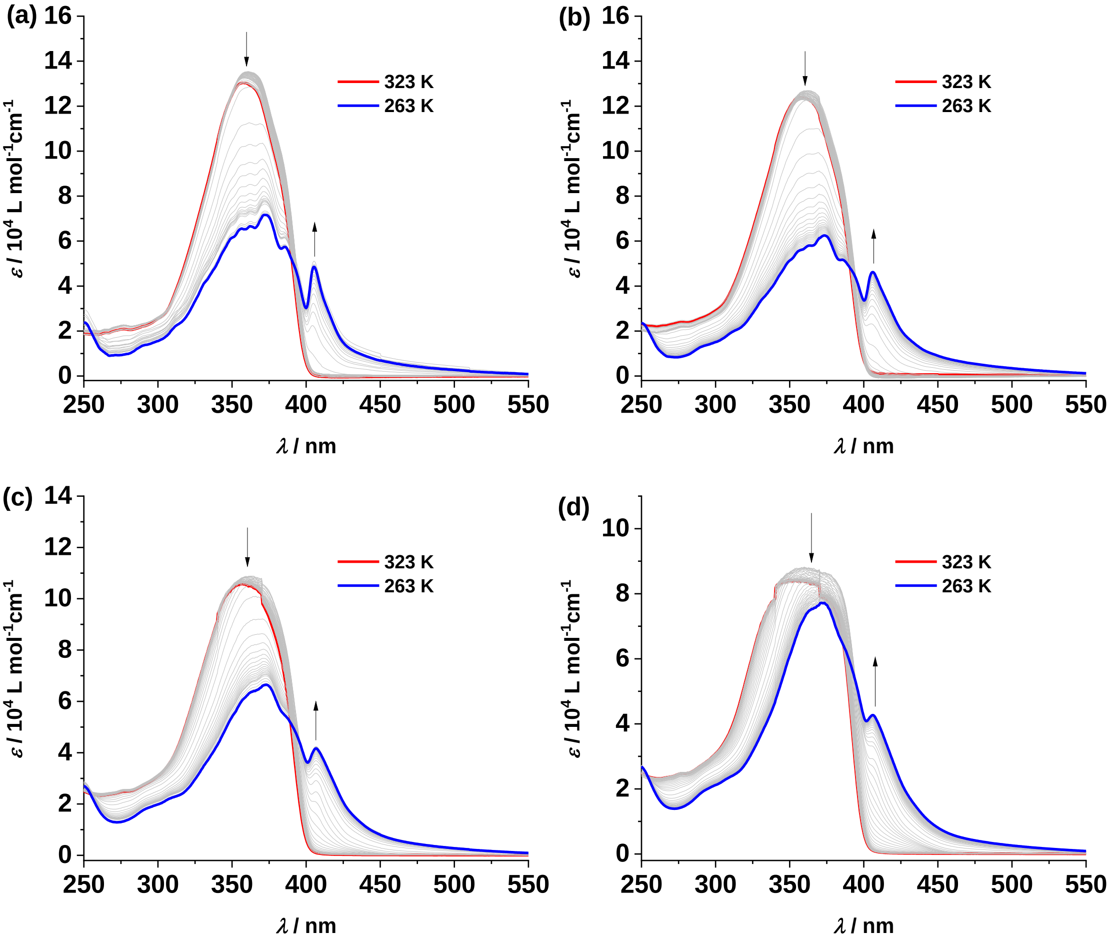

Figure S14. VT-UV/Vis spectra of **OPE6** in MCH at a) 1 mM, b) 2 mM, c) 3 mM and d) 4 mM with a cooling rate of 1 K/min.

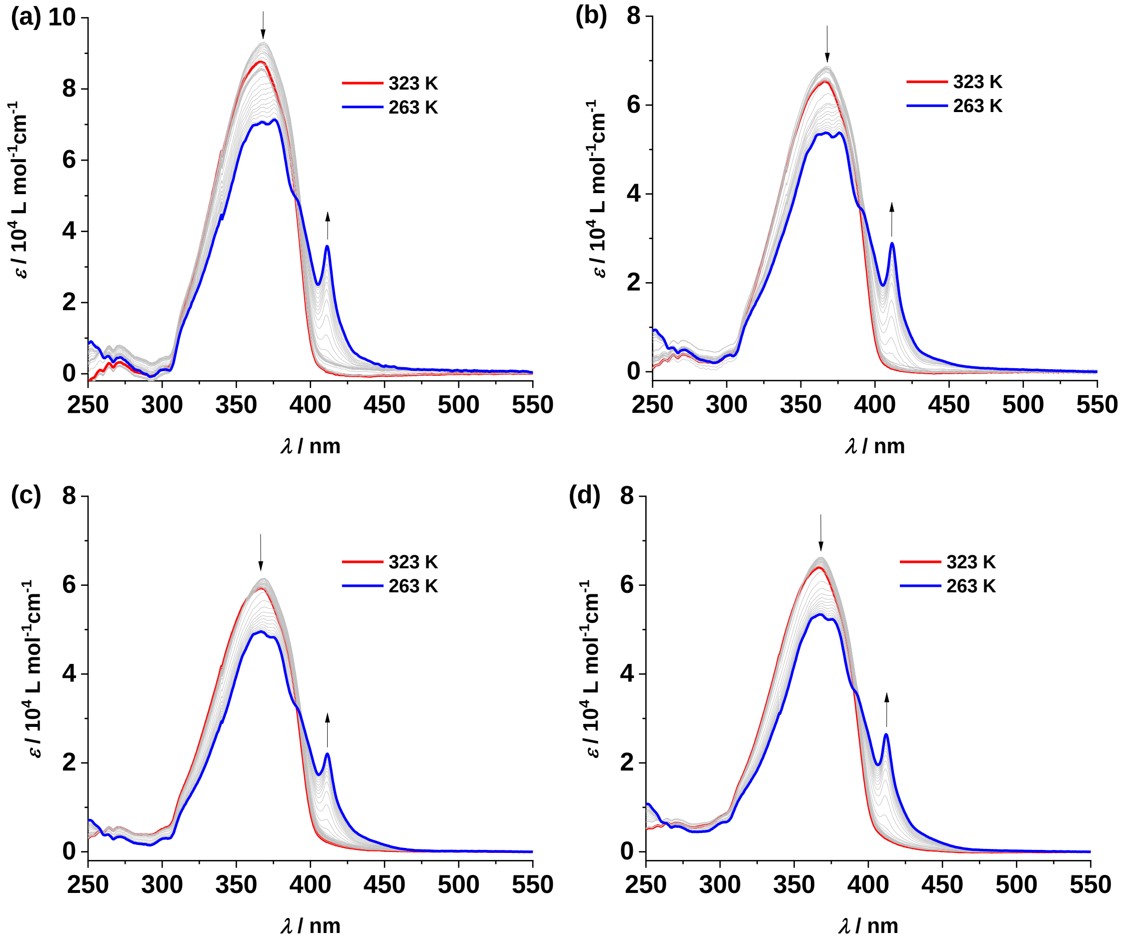

Figure S15. VT-UV/Vis spectra of **OPE7** in MCH at a) 300 µM, b) 500 µM, c) 600 µM and d) 800 µM with a cooling rate of 1 K/min.

3.1 Nucleation-Elongation Model for Cooperative Supramolecular Polymerizations

The Nucleation-Elongation model describes the equilibrium between the monomeric and the supramolecular species in a cooperative process[1]. The model is used to describe the aggregation of **OPE6** and **OPE7**, which follows a non-sigmoidal cooling curve as shown in the temperature-dependent experiments. The model extends nucleation-elongation based equilibrium models for growth of supramolecular homopolymers to the case of two monomer and aggregate types and can be applied to symmetric supramolecular copolymerizations, as well as to the more general case of nonsymmetric supramolecular copolymerizations. In a cooperative process, the polymerization occurs *via* a nucleation step followed by a nucleation step. The values T_e, $\Delta H°_{nucl}$, $\Delta H°$ and $\Delta S°$ can be obtained by a non-linear least-square analysis of the experimental melting curves. The correspondence nucleation, K_{nucl}, and elongation, K_{el}, equilibrium constants can be calculated with the following equations:

$$K_{nucl} = e^{\left[\frac{-(\Delta H^0 - \Delta H^0_{nucl}) - T\Delta S^0}{RT}\right]}$$

$$K_{el} = e^{\left[\frac{-(\Delta H^0 - T\Delta S^0)}{RT}\right]}$$

And the correspondence cooperativity factor (σ) is given by:

$$\sigma = \frac{K_{nucl}}{K_{el}} = e^{\left(\frac{\Delta H^0_{nucl}}{RT}\right)}$$

3.2 Thermodynamic Parameters

Table S2. Thermodynamic parameters for the self-assembly process of **OPE6** obtained from the global fitting of the corresponding cooling curves.

c **/ mM**	**Δ*H*⁰ / kJmol⁻¹**	**Δ*S*⁰ / kJmol⁻¹K⁻¹**	**Δ*H*⁰_N / kJmol⁻¹**	***T*_e / K**	**Δ*G*⁰_298 / kJmol⁻¹**	***K*_el**	***K*_nucl**	***σ***
**1**	-153.63 ± 2.86	-0.49 ± 0.01	-9.80 ± 0.54	281.00 ± 0.11	-8.62	1.43×10^3	21.15	1.51×10^{-2}
**2**	-153.63 ± 2.87	-0.49 ± 0.02	-9.80 ± 0.55	285.55 ± 0.12	-8.62	5.00×10^2	8.06	1.61×10^{-2}
**3**	-153.63 ± 2.88	-0.49 ± 0.03	-9.80 ± 0.56	287.35 ± 0.13	-8.62	3.33×10^2	5.51	1.65×10^{-2}
**4**	-153.63 ± 2.89	-0.49 ± 0.04	-9.80 ± 0.57	289.66 ± 0.14	-8.62	2.00×10^2	3.42	1.71×10^{-2}

Table S3. Thermodynamic parameters for the self-assembly process of **OPE7** obtained from the global fitting of the corresponding cooling curves.

c **/ μM**	**Δ*H*⁰ / kJmol⁻¹**	**Δ*S*⁰ / kJmol⁻¹K⁻¹**	**Δ*H*⁰_N / kJmol⁻¹**	***T*_e / K**	**Δ*G*⁰_298 / kJmol⁻¹**	***K*_el**	***K*_nucl**	***σ***
**300**	-109.64 ± 4.85	-0.32 ± 0.02	-10.28 ± 0.91	286.29 ± 0.24	-16.56	3.33×10^3	44.5	1.33×10^{-2}
**500**	-109.64 ± 4.86	-0.32 ± 0.03	-10.28 ± 0.92	289.50 ± 0.25	-16.56	2.00×10^3	28.0	1.40×10^{-2}
**600**	-109.64 ± 4.87	-0.32 ± 0.04	-10.28 ± 0.93	290.66 ± 0.26	-16.56	1.67×10^3	23.7	1.42×10^{-2}
**800**	-109.64 ± 4.88	-0.32 ± 0.05	-10.28 ± 0.94	292.52 ± 0.27	-16.56	1.25×10^3	18.3	1.42×10^{-2}

4 Microscopic analysis of OPE6 and OPE7

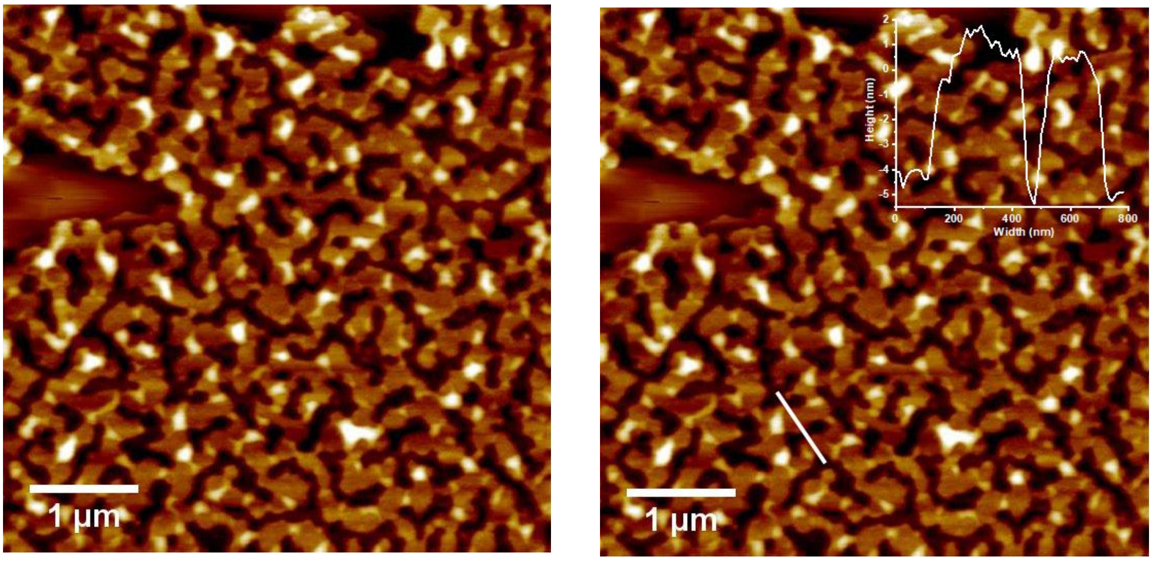

Figure S16. AFM images of **OPE6** obtained by spin coating a 250 µM solution in MCH onto mica surface.

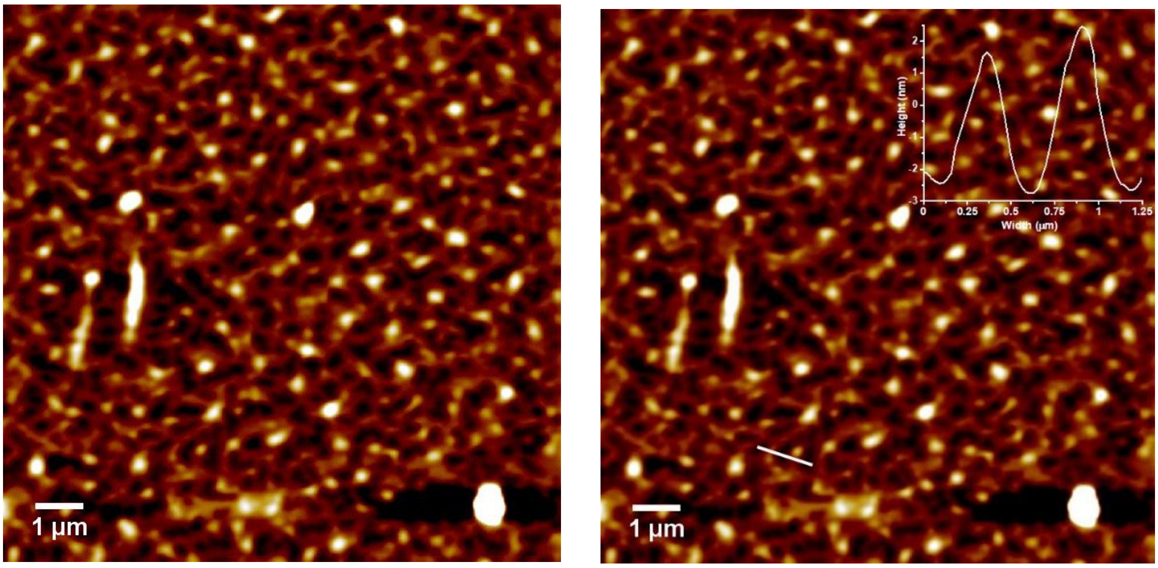

Figure S17. AFM image of **OPE7** obtained by spin coating a 250 µM solution in MCH onto mica surface.

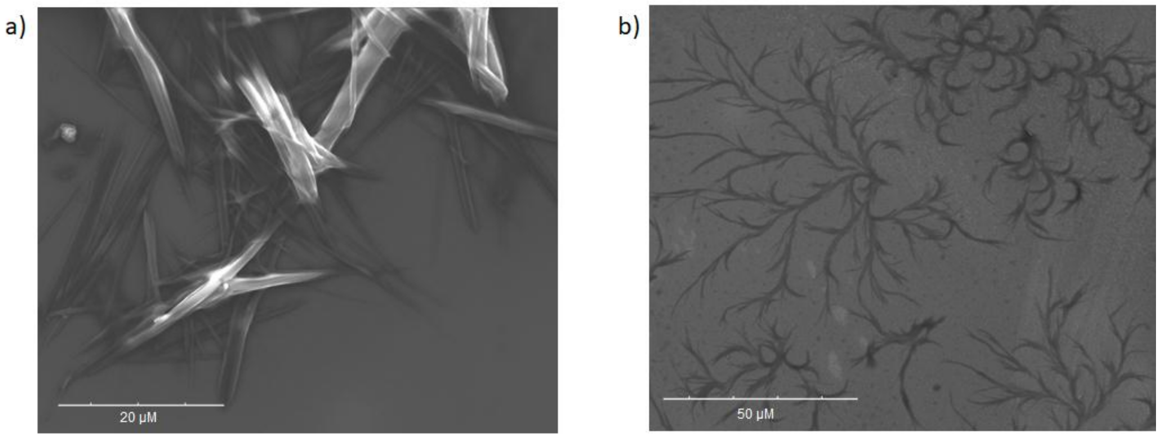

Figure S18. SEM image of a) **OPE6** and b) **OPE7** obtained by drop-casting a 250 µM solution in MCH onto silicon surface.

5 Experimental Part

5.1 Materials and Methods

General. All solvents were dried according to standard procedures. Reagents were used as purchased. All air-sensitive reactions were carried out under argon atmosphere. Flash column chromatography was performed using silica gel (Merck Silica 60, particle size 0.04-0.063 nm).

NMR measurements. ^{1}H NMR and ^{13}C NMR spectra were recorded on a *Bruker Avance 400* (^{1}H: 400 MHz; ^{13}C: 100.6 MHz) and on an *Agilent DD2 500* (^{1}H: 500 MHz; ^{13}C: 125 MHz) or an *Agilent DD2 600* (^{1}H: 600 MHz; ^{13}C: 150 MHz) at 298 K using deuterated solvents. The recorded spectra were referenced to the remaining resonance signals of the deuterated solvents. Coupling constants (J) are denoted in Hz and chemical shifts (δ) in ppm. Multiplicities are denoted as follows: s = singlet, d = doublet, t = triplet, m = multiplet. Chemical shifts are given in ppm relative to TMS (^{1}H, 0.0 ppm).

Mass Spectroscopy. ESI mass spectra were recorded on a *Bruker MicroTof* system. The signals are described by their mass/charge ratio (m/z) in u.

UV-Vis Spectroscopy. UV/Vis absorption spectra were registered using a JASCO-V770/750 spectrophotometer with a spectral bandwidth of 1.0 nm and a scan rate of 400 nm/min. All experiments were carried out using quartz cuvettes with optical paths of 1, 0.1 or 0.01 cm. For all measurements, spectroscopic grade solvents were used.

Fluorescence Spectroscopy. Fluorescence spectra were recorded on a *JASCO Spectrofluorometer FP-8500* in quartz cuvettes (SUPRASIL®, Hellma) with an optical length of 1 cm.

Atomic Force Microscopy. AFM images were recorded on a Multimode® 8 SPM System (AXS Bruker). Silicon cantilevers with a nominal spring constant of 41.0 Nm^{-1} and with resonant frequency of 300 kHz, and a typical tip radius of 7 nm (OMCL-AC160TS, Olympus) were employed. The solutions of **OPE-6** and **OPE-7** in MCH were spin-coated onto a Mica surface under 4000 rpm.

Scanning Electron Microscopy. SEM images were recorded on a Phenom Pro G6 Desktop SEM using 5 kV acceleration voltage with a secondary electron detector (SED). The solutions of **OPE6** and **OPE7** in MCH were drop-casted onto a piece of Silicon wafer.

5.2 Synthesis and Characterization of OPEs 3-7

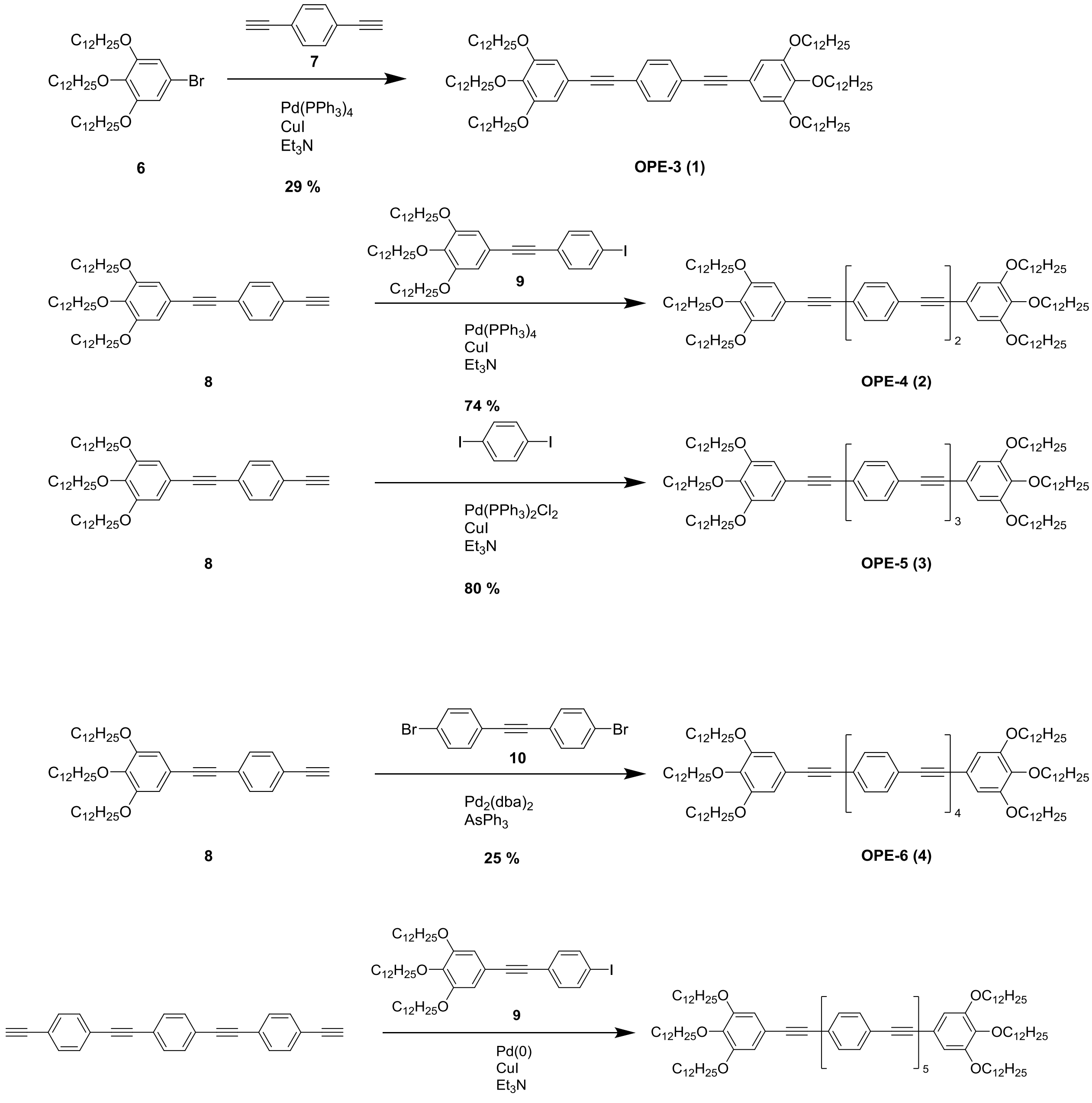

Scheme S1. Synthetic routes for **OPEs 3-7**.

Compounds **6**[2], **7**[2], **8**[3], **10**[3] and **11**[4] as well as OPE-3 (**1**)[2] and OPE-6 (**4**)[3] were synthetized following previously reported procedures and showed identical properties to those reported herein.

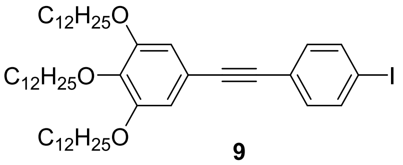

9

In a 100 mL round bottom flask, 1,2,3-tris(dodecyloxy)-5-ethynylbenzene[5] (301 mg) was dissolved in dry triethylamine (8 mL) and three Ar/vacuum cycles were flushed alternatively. In a separate flask, 1,4-diiodobenzene (217mg, 1.5 eq), $Pd(PPh_3)_2Cl_2$ (27.9 mg, 10mol%) and CuI (3.45 mg, 5mol%) were added to 20 mL dry NEt_3 and the suspension was subjected to three Ar/vacuum cycles. The initially prepared NEt_3 solution of 1,2,3-tris(dodecyloxy)-5-ethynylbenzene was added dropwise to the suspension over a period of 15-20 min. The reaction mixture was stirred at room temperature overnight. After evaporation of the solvent under reduce pressure, the crude was purified by column chromatography (Pentane/DCM 8:2) to obtain compound **9** as a white solid (182 mg, 46%).

^{1}H NMR (500 MHz, CDCl$_3$) δ (ppm) = 7.67 (d, *J* = 8.4 Hz, 2H), 7.23 (d, *J* = 8.4 Hz, 2H), 6.72 (s, 2H), 3.97 (s, 6H), 1.83 – 1.69 (m, 6H), 1.50 – 1.42 (m, 6H), 1.28 (d, *J* = 14.0 Hz, 48H), 0.88 (t, *J* = 6.9 Hz, 9H).

^{13}C NMR (125 MHz, CDCl$_3$) δ (ppm) = 153.79, 153.02, 139.32, 137.47, 132.97, 122.89, 117.20, 110.13, 110.11, 93.82, 91.20, 87.13, 73.51, 69.26, 69.14, 31.92, 31.90, 31.41, 30.30, 30.28, 30.24, 30.17, 29.73, 29.72, 29.71, 29.68, 29.64, 29.62, 29.60, 29.57, 29.37, 29.34, 29.31, 29.24, 26.07, 26.05, 26.03, 26.01, 22.68, 22.67, 14.08.

HRMS (ESI, Nanospray, CHCl$_3$/MeOH): m/z calculated for $C_{50}H_{81}IO_3Na^+$, $[M+Na]^+$ 879.51226; found 879.51239.

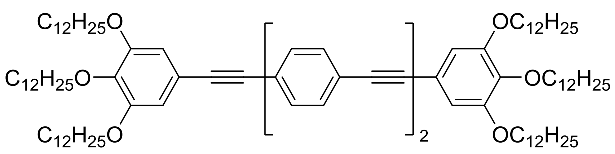

OPE-4 (2)

Compound **9** (182 mg, 0.21 mmol) and compound **8** (192 mg, 0.25 mmol), $Pd(PPh_3)_4$ (4.9 mg, 2%mol) and CuI (1.2 mg, 3%mol) were mixed in dry NEt_3 (20 mL) and the mixture was subjected to three vacuum/argon cycles. The reaction mixture was stirred at room temperature overnight. After evaporation of the solvent under reduce pressure, the crude was purified by column chromatography with Pentane/DCM (from 2:8 to 4:6) as eluent. Compound **2** was obtained as a white solid (230 mg, 74%).

^{1}H NMR (600 MHz, CDCl$_3$) δ (ppm) = 7.49 (s, 8H), 6.74 (s, 4H), 3.98 (td, *J* = 6.6, 2.1 Hz, 12H), 1.81 (p, *J* = 6.8 Hz, 8H), 1.78 – 1.69 (m, 4H), 1.47 (p, *J* = 7.8, 7.4 Hz, 12H), 1.40 – 1.19 (m, 96H), 0.88 (t, *J* = 6.9 Hz, 18H).

^{13}C NMR (150 MHz, CDCl$_3$) δ (ppm) = 153.79, 153.02, 139.32, 137.47, 132.97, 122.89, 117.20, 110.13, 110.11, 93.82, 91.20, 87.13, 73.51, 69.26, 69.14, 31.92, 31.90, 30.30, 29.73, 29.72, 29.71, 29.68, 29.64, 29.62, 29.60, 29.57, 29.37, 29.34, 29.31, 29.24, 26.07, 26.05, 22.67, 14.08.

HRMS (ESI Nanospray, $CHCl_3$/MeOH): m/z calculated for $C_{102}H_{163}0_{6,}$ $[M+H]^+$ 1485.24777; found 1485.24889.

IR (neat) solid: 2955, 2915, 2847, 2150, 1724, 1518, 1501, 1389, 1257, 1235, 1116, 833, 718, 618.

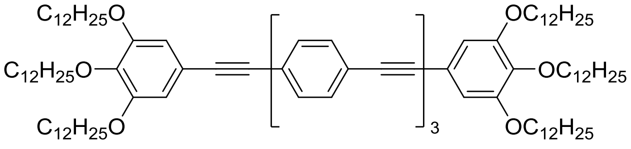

OPE-5 (3)

Compound **8** (462mg, 0.61 mmol), diiodobenzene (100mg, 0.30 mmol), $Pd(PPh_3)_2Cl_2$ (17mg, 2%mol) and CuI (6mg, 3%mol) were suspended in 8 mL NEt_3 and subjected to five vacuum/argon cycles. The reaction mixture was stirred at room temperature overnight. After NEt_3 was removed under vacuum, column chromatography of the crude was performed using Pentane/DCM (from 8:2 to 1:1) as eluent. Compound **3** (429 mg) was obtained as a yellow solid in 80% yield.

^{1}H NMR (600 MHz, $CDCl_3$) δ (ppm) = 7.51 (d, *J* = 8.7 Hz, 12H), 6.74 (s, 4H), 3.98 (td, *J* = 6.5, 2.6 Hz, 12H), 1.85 – 1.71 (m, 12H), 1.52 – 1.41 (m, 12H), 1.28 (d, *J* = 13.3 Hz, 96H), 0.88 (t, *J* = 7.0 Hz, 18H).

^{13}C NMR (150 MHz, $CDCl_3$) δ (ppm) = 153.03, 139.34, 131.56, 131.53, 131.46, 123.47, 123.06, 122.60, 117.30, 110.19, 91.88, 91.13, 90.86, 87.79, 73.55, 69.16, 31.93, 31.92, 31.42, 30.31, 30.18, 29.75, 29.74, 29.72, 29.69, 29.68, 29.65, 29.63, 29.59, 29.39, 29.36, 29.33, 26.09, 26.07, 22.69, 22.68, 14.10.

HRMS (ESI, Nanospray, $CHCl_3$/MeOH): m/z calculated for $C_{102}H_{163}0_6^+$, $[M+H]^+$ 1585.27907; found 1585.28078.

IR (neat): 2951, 2915, 2851, 1576, 1518, 1502, 1465, 1422, 1375, 1357, 1257, 1232, 1116, 991, 826, 718, 624.

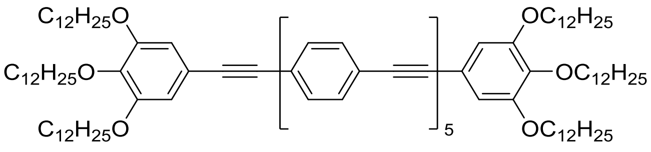

OPE-7 (5)

Compound **11** (45.2 mg, 0.14 mmol) dissolved in THF (4.5 mL) was added dropwise to a mixture containing compound **9** (253 mg, 0.29 mmol), $Pd(PPh_3)_2Cl_2$ (5.91mg, 8.42 µmol), CuI (0.88 mg, 4.62 µmol) in NEt_3 (8 mL). The reaction mixture was stirred at room temperature overnight. After removing the solvents under reduce pressure, the crude was purified using Pentane to Pentane/DCM 1:1 as eluent, affording compound **5** as a yellow solid (70%).

^{1}H NMR (500 MHz, $CDCl_3$) δ (ppm) = 7.54 – 7.47 (m, 20H), 6.74 (s, 4H), 4.00 – 3.94 (m, 12H), 1.85 – 1.70 (m, 12H), 1.47 (td, *J* = 8.5, 4.8 Hz, 12H), 1.27 (s, 96H), 0.89 (s, 18H).

^{13}C NMR (125 MHz, $CDCl_3$) δ (ppm) = 153.05, 139.36, 131.60, 131.59, 131.55, 131.48, 123.50, 123.14, 123.07, 123.01, 122.60, 117.31, 110.20, 91.91, 91.18, 87.80, 73.56, 69.18, 31.95, 31.93, 30.33, 29.76, 29.75, 29.74, 29.71, 29.67, 29.65, 29.60, 29.40, 29.37, 29.34, 26.11, 26.09, 22.70, 22.70, 14.12.

HRMS (ESI, Nanospray $CHCl_3$/MeOH): m/z calculated for $C_{126}H_{175}O_6^+$, $[M+H]^+$ 1785.34167; found 1785.33977.

IR (neat): 2980, 2919, 2851, 2355, 1572,1515, 1461, 1414, 1378, 1353, 1253, 1232, 1116, 955, 830, 720.

5.3 ^{1}H and ^{13}C NMR Spectra

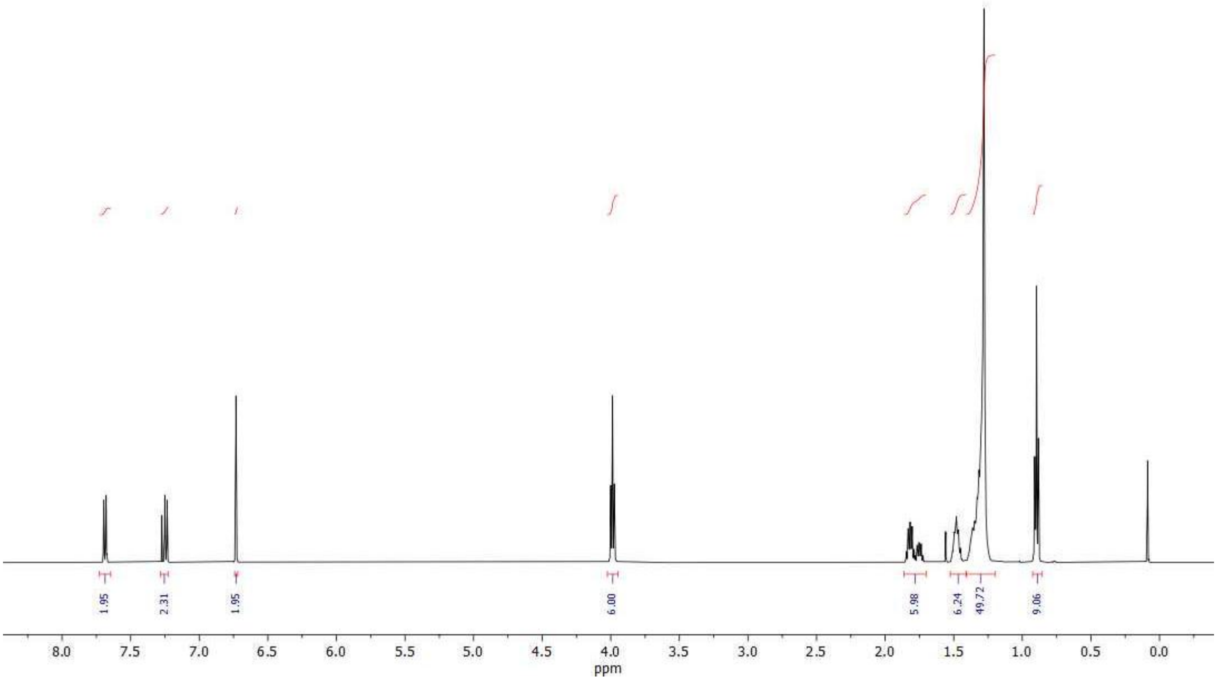

Figure S19. ^{1}H NMR spectrum for compound **9** ($CDCl_3$, 500 MHz, 298 K).

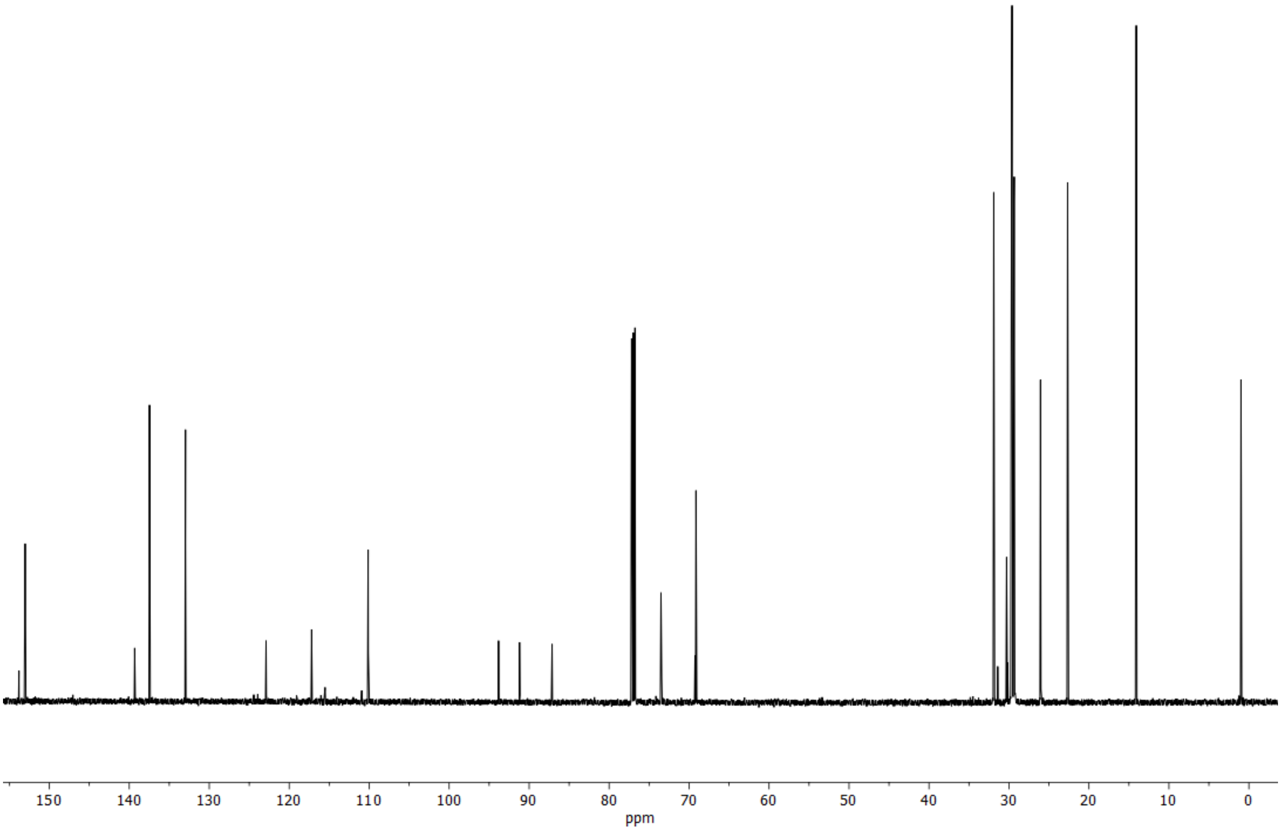

Figure S20. ^{13}C NMR spectrum for compound **9** ($CDCl_3$, 125 MHz, 298 K).

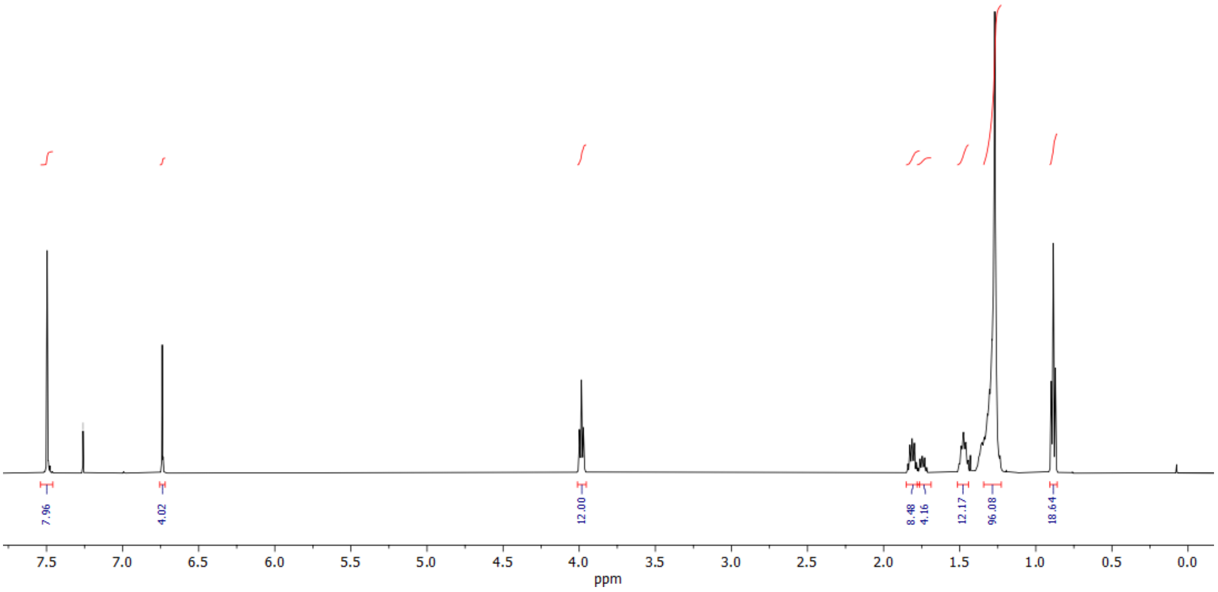

Figure S21. ^{1}H NMR spectrum for compound **2 (OPE4)** ($CDCl_3$, 600 MHz, 298 K).

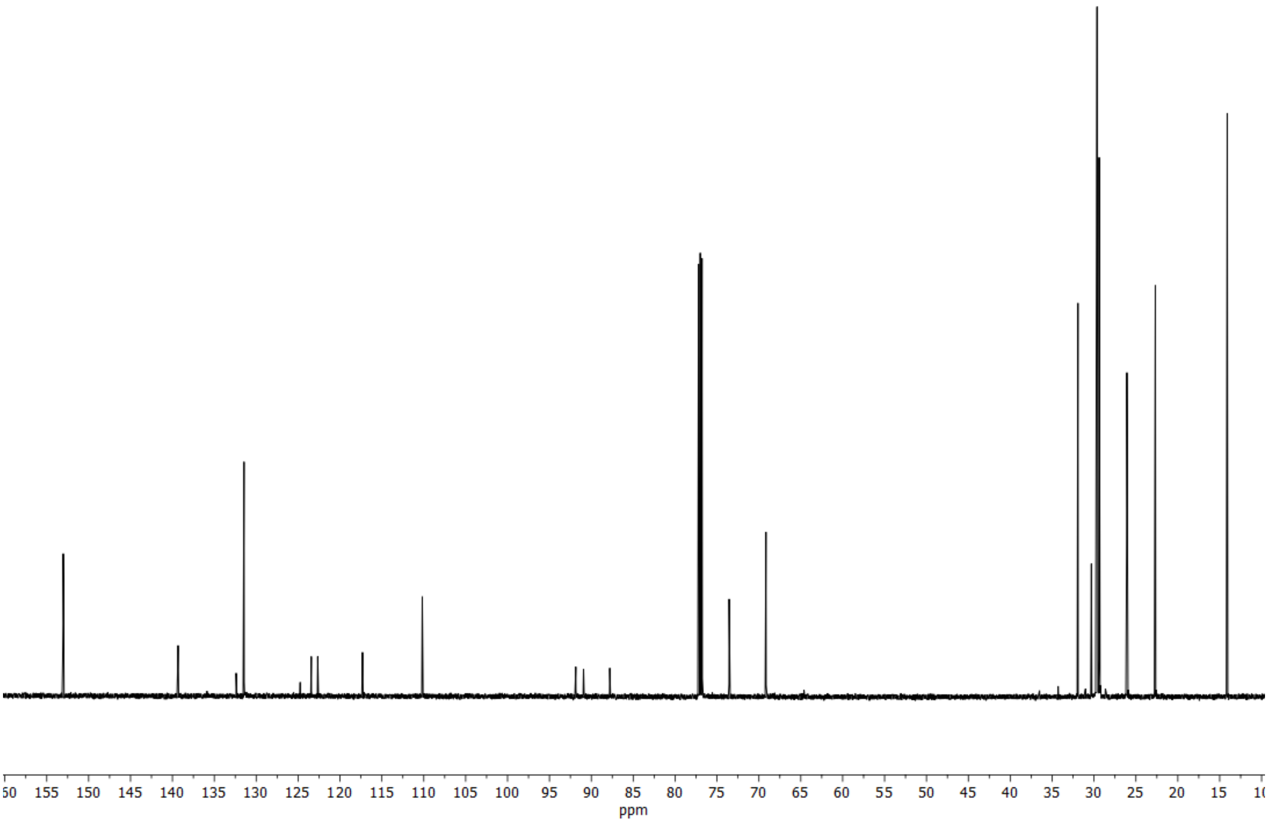

Figure S22. ^{13}C NMR spectrum for compound **2 (OPE4)** ($CDCl_3$, 150 MHz, 298 K).

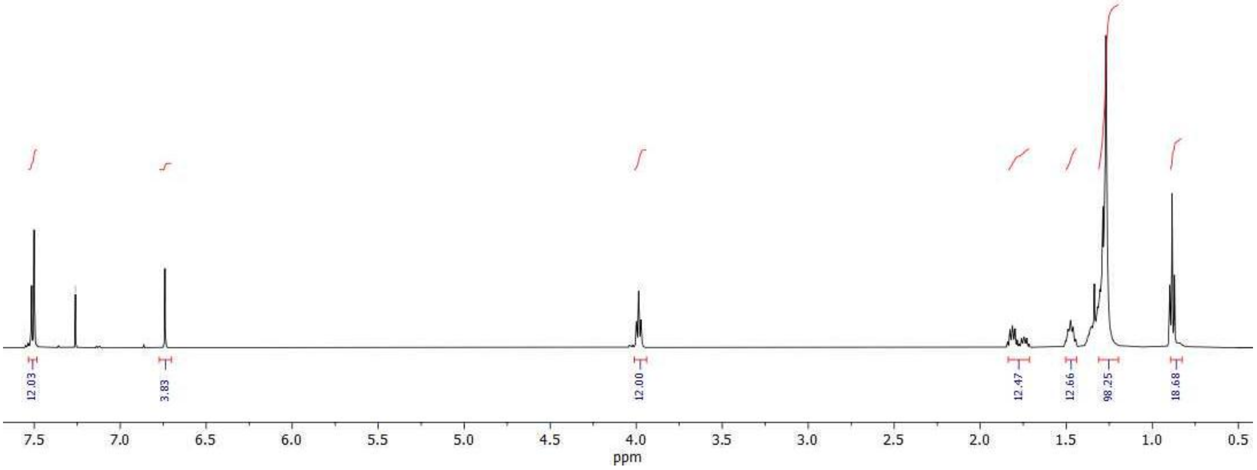

Figure S23. ^{1}H NMR spectrum for compound **3 (OPE5)** ($CDCl_3$, 600 MHz, 298 K).

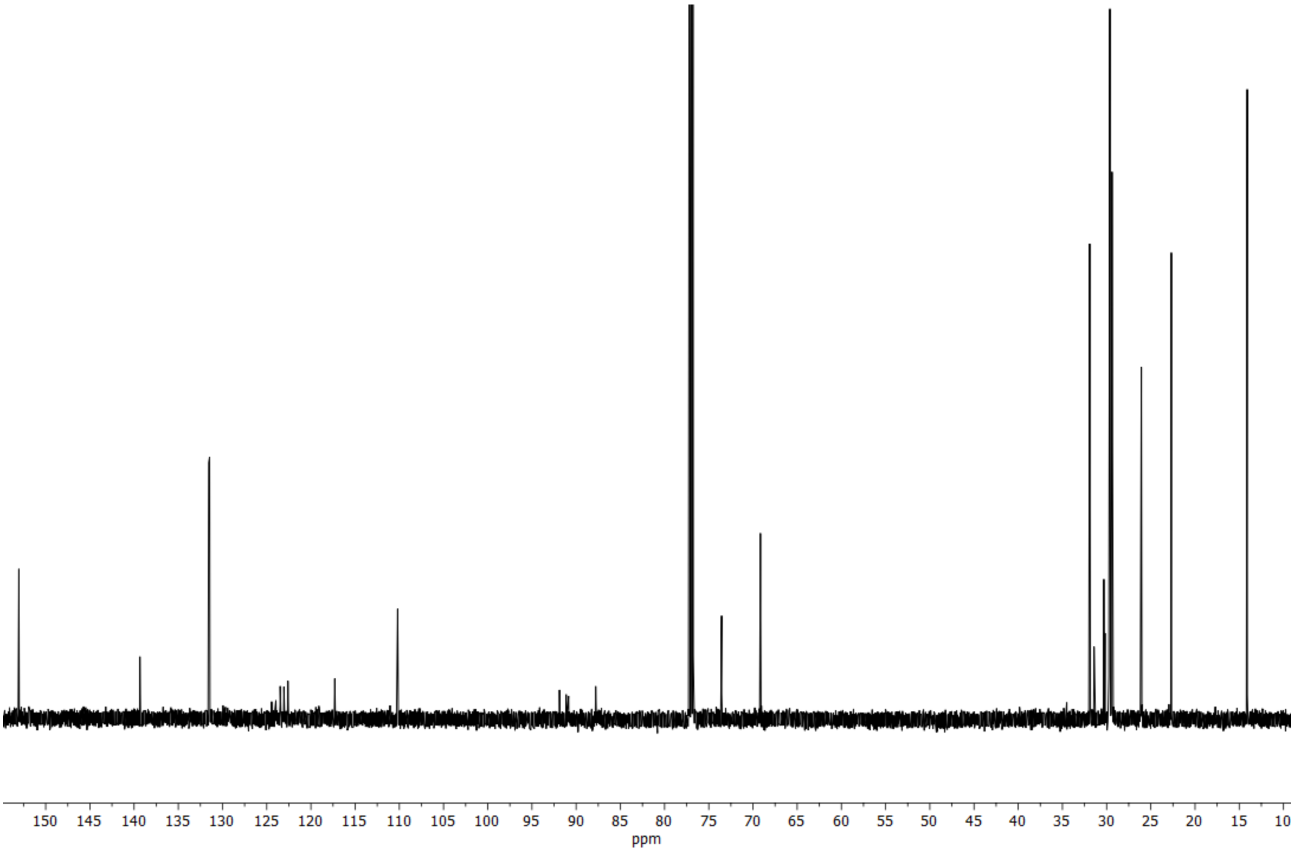

Figure S24. ^{13}C NMR spectrum for compound **3 (OPE5)** ($CDCl_3$, 150 MHz, 298 K).

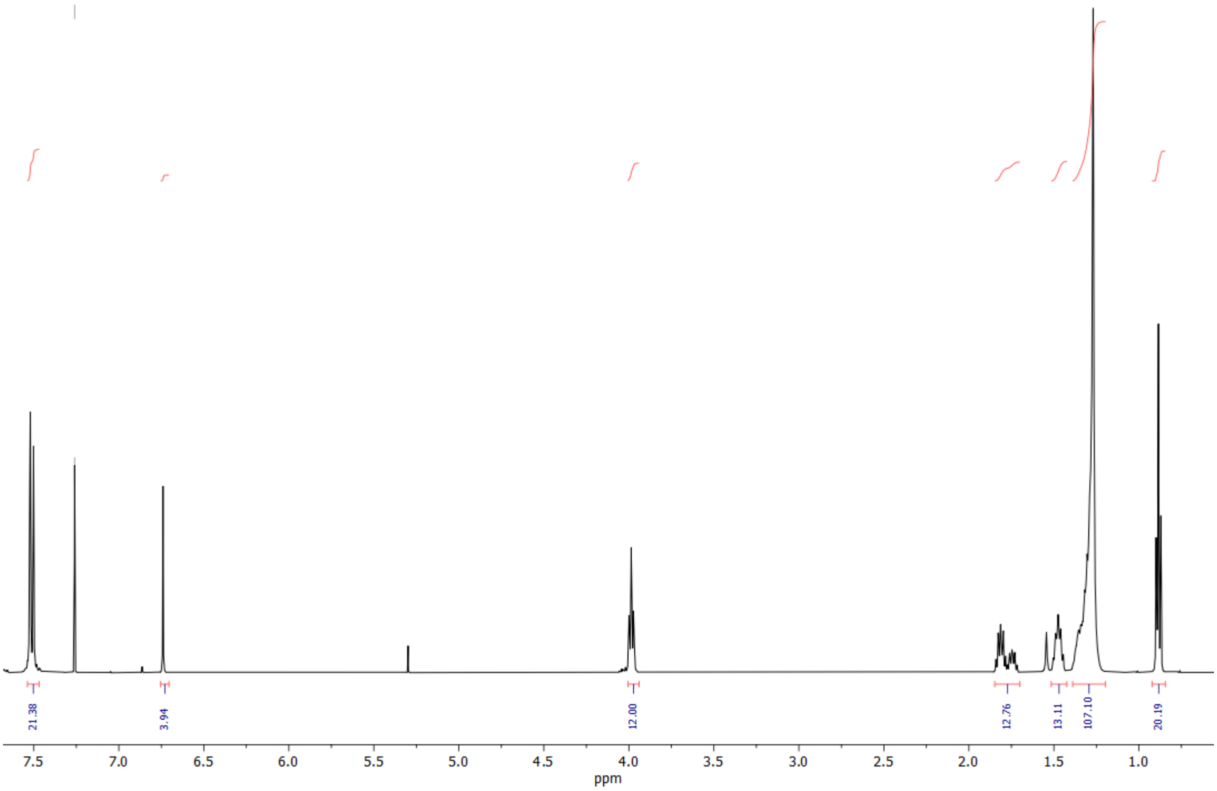

Figure S25. ^{1}H NMR spectrum for compound **5 (OPE7)** ($CDCl_3$, 500 MHz, 298 K).

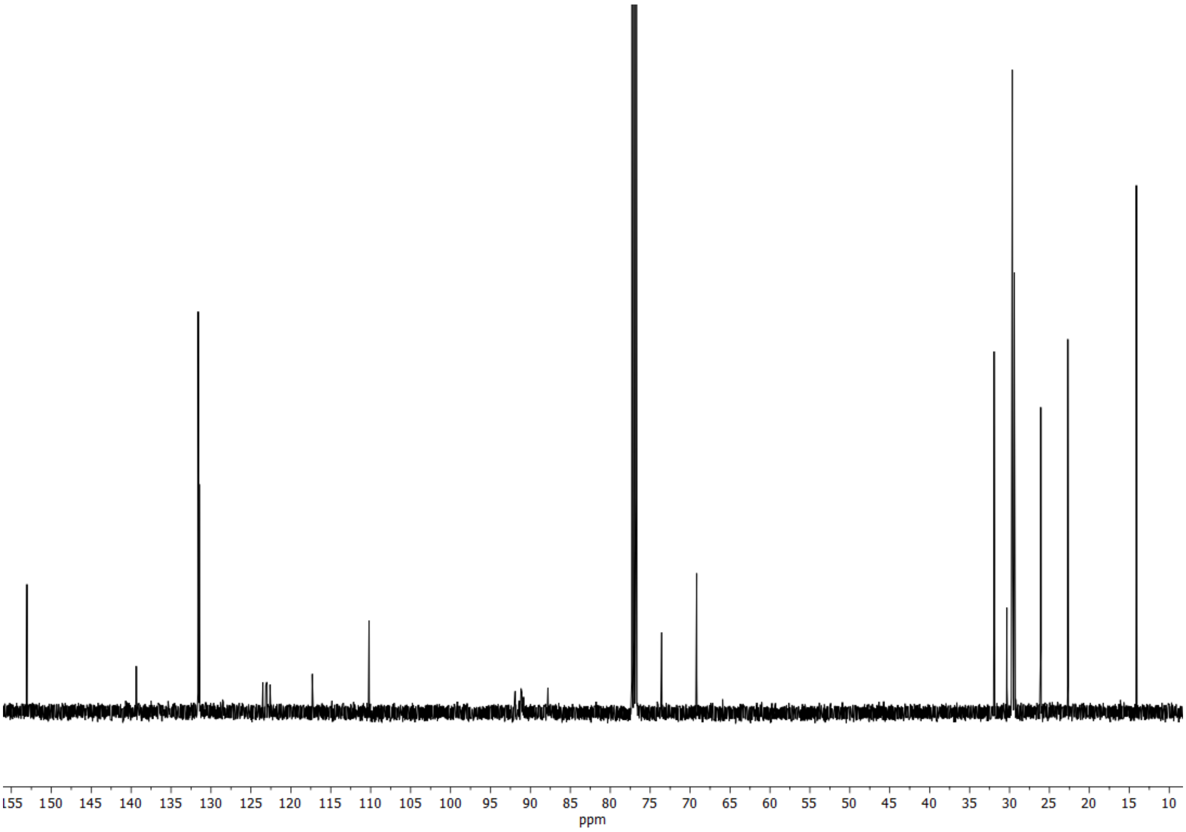

Figure S26. ^{13}C NMR spectrum for compound **5 (OPE7)** ($CDCl_3$, 125 MHz, 298 K).

6 References

1. a) H. M. M. ten Eikelder, A. J. Markwoort, T. F. A. De Greef, P. A. J. Hilbers, *J. Phys. Chem. B,* 2012, **116**, 5291-5301; b) A. J. Maarkvort, H. M. M. ten Eikelder, P. J. J. Hilbers, T. F. A. De Greef, E. W. Meijer, *Nat. Commun.,* 2011, **2**, 509-517.

2. M. J. Mayoral, C. Rest, J. Schellheimer, V. Stepanenko, G. Fernández, *Chem. Eur. J.,* 2012, **18**, 15607-15611.

3. M. J. Mayoral, C. Rest, V. Stepanenko, J. Schellheimer, R. Albuquerque, G. Fernández, *J. Am. Chem. Soc.*, 2013, **135**, 2148-2151.

4. T. Rudolph, N. K. Allampally, G. Fernández, F. H. Schacher, *Chem. Eur. J.*, 2014, **20**, 13871-13875.

5. M. Lübtow, I. Helmers, V. Stepanenko, R. Albuquerque, T. B. Marder, G. Fernández, *Chem. Eur.J.*, 2017, **23**, 6198-6205.
